# miR-15/16 Restrain Memory T Cell Differentiation, Cell Cycle, and Survival

**DOI:** 10.1016/j.celrep.2019.07.064

**Published:** 2019-08-20

**Authors:** John D. Gagnon, Robin Kageyama, Hesham M. Shehata, Marlys S. Fassett, Darryl J. Mar, Eric J. Wigton, Kristina Johansson, Adam J. Litterman, Pamela Odorizzi, Dimitre Simeonov, Brian J. Laidlaw, Marisella Panduro, Sana Patel, Lukas T. Jeker, Margaret E. Feeney, Michael T. McManus, Alexander Marson, Mehrdad Matloubian, Shomyseh Sanjabi, K. Mark Ansel

**Affiliations:** 1 Biomedical Sciences Graduate Program, University of California, San Francisco, San Francisco, CA 94143, USA; 2 Department of Microbiology and Immunology, University of California, San Francisco, San Francisco, CA 94143, USA; 3 Sandler Asthma Basic Research Center, University of California, San Francisco, San Francisco, CA 94143, USA; 4 Virology and Immunology, Gladstone Institutes, San Francisco, CA 94158, USA; 5 Department of Dermatology, University of California, San Francisco, San Francisco, CA 94143, USA; 6 Department of Medicine, University of California, San Francisco, San Francisco, CA 94143, USA; 7 Diabetes Center, University of California, San Francisco, San Francisco, CA 94143, USA; 8 Diabetes Center and Department of Medicine, University of California, San Francisco, San Francisco, CA 94143, USA; 9 Department of Pathology, University of California, San Francisco, San Francisco, CA 94143, USA; 10 Present address: Department of Biomedicine, Basel University Hospital and University of Basel, 4031 Basel, Switzerland; 11 Transplantation Immunology & Nephrology, Basel University Hospital, 4031 Basel, Switzerland; 12 Lead Contact

## Abstract

Coordinate control of T cell proliferation, survival, and differentiation are essential for host protection from pathogens and cancer. Long-lived memory cells, whose precursors are formed during the initial immunological insult, provide protection from future encounters, and their generation is the goal of many vaccination strategies. microRNAs (miRNAs) are key nodes in regulatory networks that shape effective T cell responses through the fine-tuning of thousands of genes. Here, using compound conditional mutant mice to eliminate miR-15/16 family miRNAs in T cells, we show that miR-15/16 restrict T cell cycle, survival, and memory T cell differentiation. High throughput sequencing of RNA isolated by cross-linking immuno-precipitation of AGO2 combined with gene expression analysis in miR-15/16-deficient T cells indicates that these effects are mediated through the direct inhibition of an extensive network of target genes within pathways critical to cell cycle, survival, and memory.

## In Brief

Coordinate control of T cell proliferation, survival, and differentiation are essential for effective cell-mediated adaptive immunity. Gagnon et al. define roles for the miR-15/16 family of microRNAs in restricting T cell cycle and long-lived memory T cell accumulation through the direct inhibition of a very large network of target mRNAs.

## Graphical Abstract

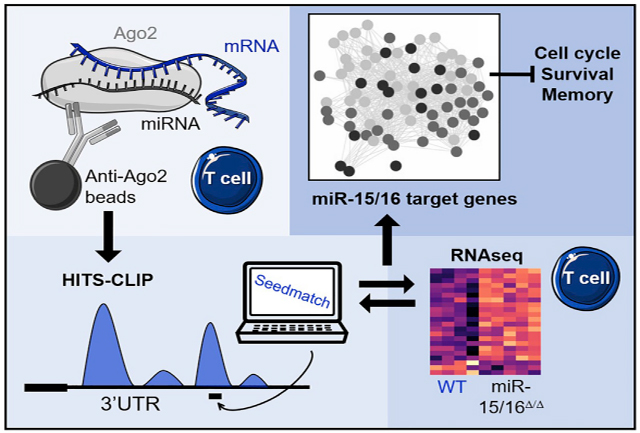

## INTRODUCTION

Regulation of T cell proliferation, survival, and differentiation is vital for effective immunity. In response to immunological challenges, naive antigen-specific T cells expand rapidly and undergo massive gene expression changes. As many as 50% of these changes are mediated post-transcriptionally (Cheadle et al., 2005). Within the first division, responding CD8^+^ T cells acquire sustained gene expression programs that lead to their differentiation into appropriately proportionate populations of terminal effector (TE) and memory precursor (MP) cells, identified by the expression of killer cell lectin-like receptor subfamily G member 1 (KLRG1) and IL-7 receptor alpha (*Il7r*/CD127), respectively (Kaech et al., 2002, 2003; Kakaradov et al., 2017; Sarkar et al., 2008). Effector T cells are important for pathogen or tumor clearance. MP cells form a long-lived pool of memory cells capable of rapidly responding to subsequent encounters with the same antigen.

microRNAs (miRNAs) are short non-coding RNAs that mediate post-transcriptional regulation, predominantly via Watson-Crick base pairing to 3′ untranslated regions (UTRs) of target mRNAs (Agarwal et al., 2015). miRNAs play key roles in the differentiation and functional characteristics of memory T cells (Ban et al., 2017; Baumjohann and Ansel, 2013; Chen et al., 2017; Guan et al., 2018; Khan et al., 2013; Moffett et al., 2017; Pua et al., 2016; Steiner et al., 2011; Wells et al., 2017). While the effects of miRNAs on the downregulation of individual targets are often less than two-fold, through coordinated tuning of gene networks, their overall biological effects can be profound.

The miR-15/16 family of miRNAs functions as tumor suppressors. Deletions of the *Dleu2/Mirc30* locus, which encodes miR-15a and miR-16–1, occur in more than 50% of human chronic lymphocytic leukemia (CLL) cases (Calin et al., 2002), and targeted deletion of these miRNAs in mice induces a CLL-like indolent B lymphocyte proliferative disease (Klein et al., 2010). miR-15/16 restrict the proliferation of B cells through the direct targeting of numerous cell-cycle- and survival-associated genes, including *Ccnd1, Ccne1*, *Cdk6,* and *Bcl2* (Liu et al., 2008). In addition to *Mirc30,* T cells strongly express *Mirc10* and its two mature miRNA products, miR-15b and miR-16–2. Patients with T cell lymphoblastic lymphoma/leukemia (T-LBL/ALL) exhibiting lower-than-median expression levels of miR-16 exhibit a worse prognosis, suggesting a similar role for miR-15/16 in T cells (Xi et al., 2013). miR-15/16 has also been implicated in T cell anergy, regulatory T cell (Treg) induction, Treg/Th17 balance, and tumor-infiltrating T cell activation (Marcais et al., 2014; Singh et al., 2015; Wu et al., 2016; Yang et al., 2017). However, the requirements for miR-15/16 in T cell development, proliferation, survival, and differentiation remain unknown.

We generated mice with conditional inactivation of both *miR-15a/16–1/Mirc30* and *miR-15b/16–2/Mirc10* in T cells (*miR-15/16*^*Δ/Δ*^). miR-15/16 restricted T cell proliferation, limited T cell survival *ex vivo,* and directly targeted numerous cell-cycle- and survival-associated genes. Deletion of miR-15/16 in T cells did not result in overt lymphoproliferative disease. Instead, *miR-15/16*^*Δ/Δ*^ mice selectively accumulated memory T cells, and miR-15/16 restricted the differentiation of MP cells in response to the lymphocytic choriomeningitis virus (LCMV). Rather than working through any one critical target, miR-15/16 physically interacted with and repressed the expression of a surprisingly broad network of memory-associated genes.

## RESULTS

### miR-15/16 Are Dynamically Regulated during T Cell Responses

**Figure 1.**
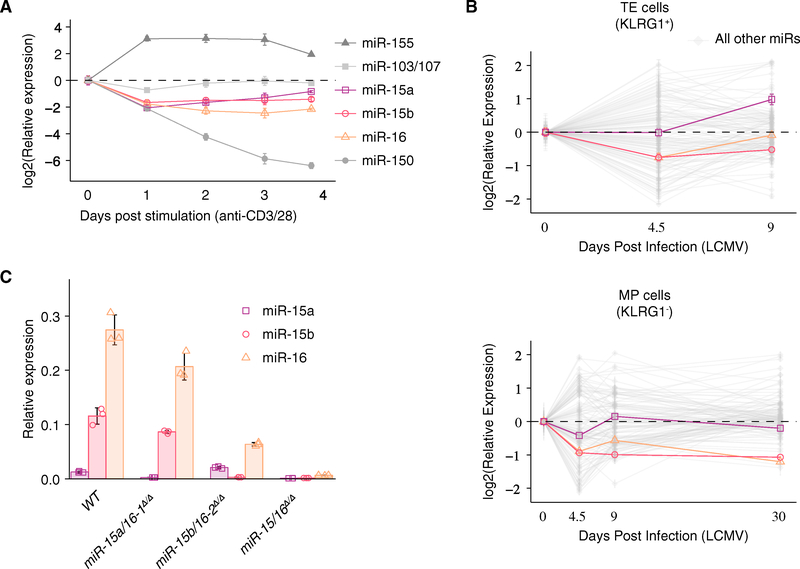
miR-15/16 Are Dynamically Regulated during T Cell Responses (A) qPCR of miRNA expression within CD4^+^ T cells in response to *in vitro* stimulation with anti-CD3 and anti-CD28 for 3 days followed by 1 day resting v (n = 6 biological replicates from two independent experiments). (B) Time course miRNA microarray of CD8^+^ TE and MP cells after infection with LCMV (n = 3 biological replicates from one experiment). (C) qPCR of miRNAs purified from naive CD4^+^ T cells of the indicated genotypes (n = 3 biological replicates from one experiment). See also Figure S1.

Activated T cells rapidly reset their mature miRNA repertoire through an increased turnover of the miRNA-induced silencing complex (miRISC) and transcriptional regulation of miRNA precursors (Bronevetsky et al., 2013). Consistent with this prior report, miR-15a, miR-15b, and miR-16 were substantially downregulated over a 4-day course of CD4^+^ T cell activation *in vitro* (Figure 1A). miR-155 (upregulated), miR-103/107 (transiently downregulated), and miR-150 (downregulated) also behaved as expected. To assess expression kinetics in a physiologically relevant context, we re-analyzed published data from CD8^+^ TE and MP cells sorted from LCMV-infected mice (Khan et al., 2013). miR-15/16 were downregulated in both TE and MP cells (Figure 1B). In MP cells, miR-15b and miR-16 downregulation was sustained for at least 30 days post-infection (p.i.), placing these miRNAs among the most downregulated during memory T cell formation. miR-15a expression recovered to naive T cell levels by 30 days p.i. in MP cells (Figure 1B). However, miR-15a accounts for <10% of the total miR-15/16 family miRNAs in resting CD4^+^ T cells (Figure 1C). These results suggest that limiting the expression of miR-15/16 may be an important component of the gene expression program initiated by T cell activation and sustained among memory CD8^+^ T cells.

### T Cell-Specific Inactivation of Both miR-15/16 Gene Loci

miR-15/16 clusters occur in two genomic locations. The *miR-15a/16–1* cluster (*Mirc30*) resides intronic to the long non-coding RNA, *Dleu2*, on chromosome 14 in mice (13 in humans), and *miR-15b/16–2* (*Mirc10*) resides intronic to *Smc4* on chromosome 3 within the genomes of mice and humans. Previous studies of these miRNAs have been restricted to genetic ablation of either one of these clusters independently, overexpression, or transient inhibition. However, cells with CD4-Cre-mediated deletion of either one of these clusters independently (*miR-15a/16–1*^*Δ/Δ*^ or *miR-15b/16–2*^*Δ/Δ*^ T cells) retained high levels of miR-16 expression, whereas the removal of both clusters (*miR-15/16*^*Δ/Δ*^) effectively abrogated miR-15 and miR-16 expression (Figure 1C).

T cell-specific deletion of both miR-15/16 clusters revealed subtle effects on T cell accumulation in primary and secondary lymphoid tissues without affecting total cellularity (Figures S1A–S1C). These effects were cell intrinsic, as they were also observed among *miR-15/16*^*Δ/Δ*^ T cells within mixed bone marrow chimeric mice (Figure S1D). Mice with B cell-specific deletion of miR-15a/16–1 develop a delayed clonal lymphocytosis (Klein et al., 2010). *miR-15/16*^*Δ/Δ*^ animals were examined after aging over 1.5 years with no apparent lymphoproliferative disease, lymphoma, or leukemia (data not shown). These findings demonstrate that miR-15/16 restrict the accumulation of T cells in specific-pathogen-free animals without causing disease.

### miR-15/16 Bind and Regulate a Large Network of Direct Target RNAs in T Cells

**Figure 2.**
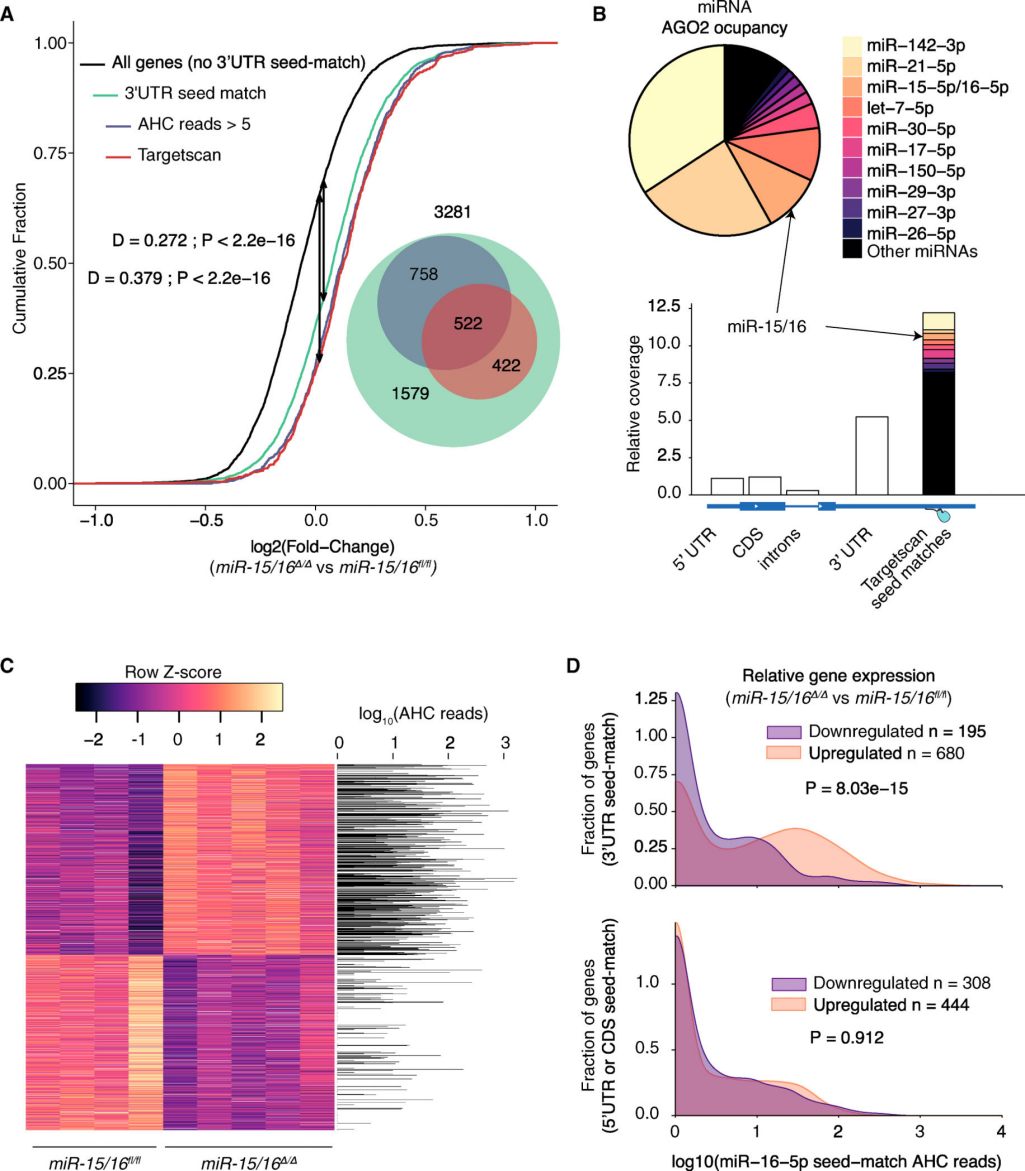
miR-15/16 Bind and Regulate a Large Network of Direct Target RNAs in T Cells (A) Cumulative density plot depicting global expression by RNA sequencing as a ratio of the fold change between *miR-15/16*^*Δ/Δ*^ (n = 5 biological replicates) and *miR-15/16*^*fl/fl*^ (n = 4 biological replicates) resting CD4^+^ T cells for all genes without a 7-mer or 8-mer miR-15/16 3′UTR seed match (black), genes with a 7-mer or 8-mer miR-15/16 3′UTR seed match (green), genes with a 7-mer or 8-mer miR-15/16 3′UTR seed match and AHC read depth ≥5 (blue), and genes classified as targets of miR-15/16 by TargetScan 7.0 (red) (AHC reads represent the combined depth of n = 10 independent immunoprecipitations). (B) Relative abundance of miRNAs bound by AGO2 (top) and AHC coverage across transcriptomic locations relative to region length (bottom). (C) Heatmap of genes with a p value ≤ 0.05 plotted alongside a bar graph of AHC read depth at miR-15/16 seed matches for each gene at which they occur. (D) Comparison of AHC reads between genes that are downregulated and upregulated (p ≤ 0.05) in *miR-15/16*^*Δ/Δ*^ cells among genes with seed matches in the 3′UTR (top) or 5′UTR/CDS (bottom) (Mann-Whitney U test). See also Figure S2.

The RNA sequencing (RNA-seq) comparison of *miR-15/16*^*fl/fl*^ and *miR-15/16*^*Δ/Δ*^ T cells showed that genes predicted to be targeted by miR-15/16 by TargetScan 7.1 (Agarwal et al., 2015) were more likely to be upregulated in *miR-15/16*^*Δ/Δ*^ T cells, compared to all expressed genes lacking a miR-15/16 seed match in their 3′UTR (Figure 2A). The full set of genes with 3′UTR seed matches was also enriched for upregulation in *miR-15/16*^*Δ/Δ*^ cells. This set contains three times as many putative target genes as the restricted TargetScan subset (3281 versus 944), but also many more false-positive targets.

To further enrich for genes directly targeted in T cells, we performed AGO2 high-throughput sequencing of RNAs isolated by crosslinking immunoprecipitation (AHC) to globally map AGO2-miRNA complex binding to target RNAs (Chi et al., 2009). Mature miRNAs accounted for 37% of all AHC reads (Figure S2A), and among these, miR-15/16 was the third most abundant family (Figure 2B, top). Non-miRNA AHC relative coverage was highly enriched at TargetScan-predicted miRNA binding sites, and again miR-15/16 seed matches were the third most abundant (Figure 2B, bottom). Indeed, there was a significant correlation between the density of AHC reads mapping to all miRNAs and their corresponding seed matches (Figure S2B).

Restricting the list of putative miR-15/16 targets to the 1280 genes with 3′UTR seed matches corresponding with AHC read depth ≥5 enriched for genes upregulated in *miR-15/16*^*Δ/Δ*^ cells equally as well as TargetScan (Figure 2A). Supporting the validity of this approach and the large number of detected direct miR-15/16 target mRNAs expressed in T cells, differentially expressed genes (p ≤ 0.05) upregulated in *miR-15/16*^*Δ/Δ*^ cells were frequently occupied by AGO2 at miR-15/16 seed matches, as compared with genes downregulated in *miR-15/16*^*Δ/Δ*^ cells (Figure 2C). Furthermore, upregulated genes with 3′UTR seed matches (n = 680) had significantly greater AHC read depth than downregulated genes with 3′UTR seed matches (n = 195) (Figure 2D, top). In contrast, upregulated genes in *miR-15/16*^*Δ/Δ*^ cells containing 5′UTR or coding sequence (CDS) but not 3′UTR seed matches had no enrichment for AGO2 binding, compared with downregulated genes (Figure 2D, bottom). Together, these data indicate that miR-15/16 directly binds a large network of target genes in T cells.

### miR-15/16 Directly Target Cell-Cycle-Associated Genes and Restrict Accumulation of Antigen-Specific T Cells in Response to LCMV Infection

**Figure 3.**
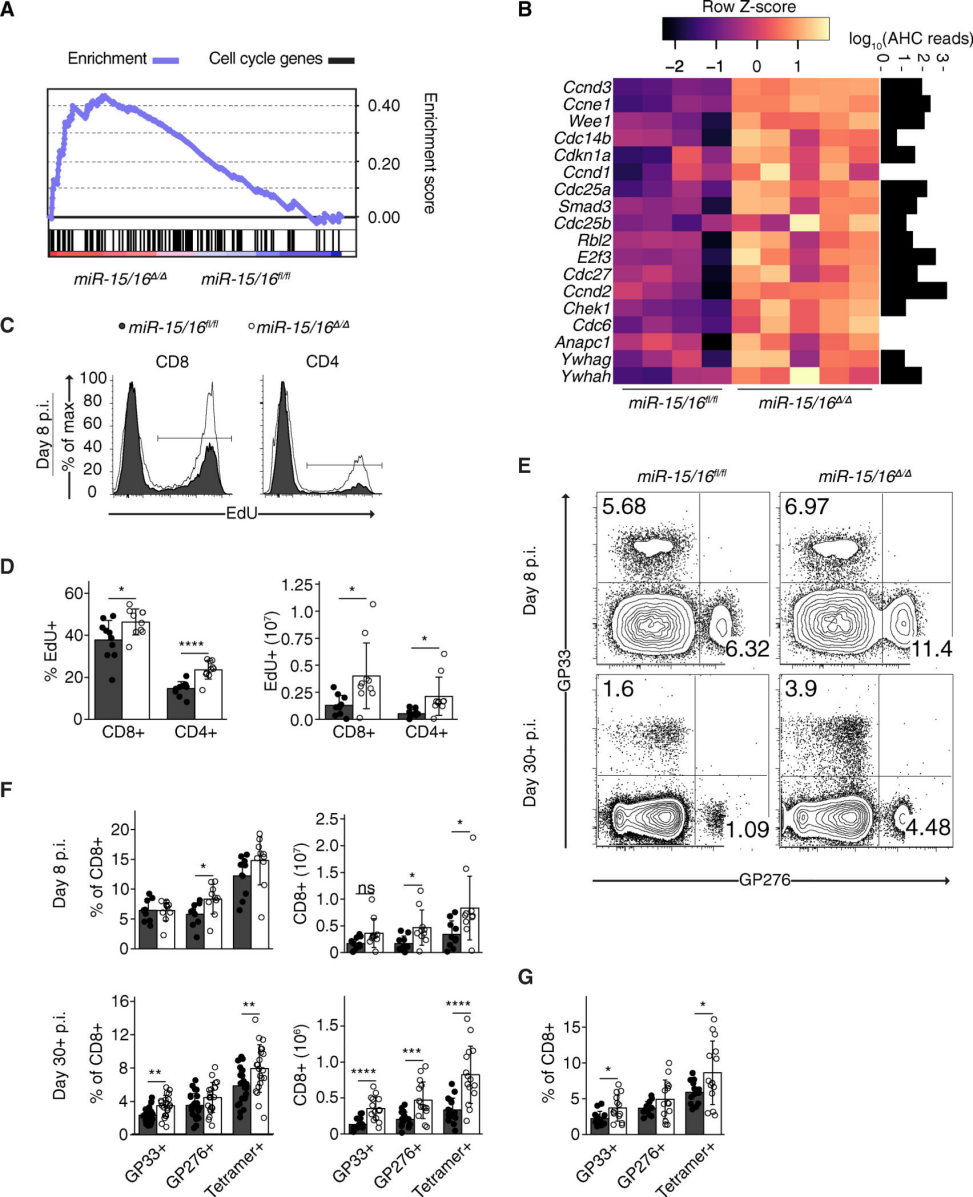
miR-15/16 Directly Target Cell-Cycle-Associated Genes and Restrict Accumulation of Antigen-Specific T Cells in Response to LCMV Infection (A) Gene set enrichment analysis of all expressed genes (normalized read depth ≥5). Plotted are the enrichment curve for and positional location of each target in the KEGG Cell Cycle gene set arrayed in ranked order from most upregulated to most downregulated (left to right) in *miR-15/16*^*Δ/Δ*^ (enrichment score (ES) = 0.4354147; nominal p value < 0.001; false discovery rate [FDR] q value = 0.088). (B) Heatmap of KEGG Cell Cycle genes with miR-15/16 seed matches in their 3′UTRs plotted alongside a bar graph of AHC read depth at miR-15/16 seed matches for each gene at which they occur. (C) Flow cytometry of EdU-labeled CD8^+^ and CD4^+^ T cells 8 days p.i. (D) Quantification of frequencies (left) and absolute numbers (right) of EdU^+^ CD8^+^ and CD4^+^ T cells 8 days p.i. with LCMV (n = 10 biological replicates representative of two independent experiments, two-tailed t test). (E) Flow cytometry of antigen-specific T cells. (F) Quantification of frequencies (left) and absolute numbers (right) of antigen-specific CD8^+^ T cells (n = 10 biological replicates representative of two independent experiments, two-tailed t test). (G) Quantification of frequencies of antigen-specific CD8^+^ T cells in mixed bone marrow chimeras (n ≥ 8 biological replicates, representative of ≥3 independent experiments, two-tailed t test). *p < 0.05; **p < 0.01; ***p < 0.001; ****p < 0.0001. See also Figure S3.

Gene set enrichment analysis identified KEGG Cell Cycle as a major signature of gene expression in *miR-15/16*^*Δ/Δ*^ cells (Figure 3A). Strikingly, all differentially expressed genes (p ≤ 0.05) within the KEGG Cell Cycle gene set containing miR-15/16 seed matches were upregulated in *miR-15/16*^*Δ/Δ*^ cells (Figure 3B). All but three of these genes had considerable AHC reads at miR-15/16 seed matches, suggesting that they were directly targeted by miR-15/16.

To test whether miR-15/16 restrict the T cell response to antigen stimulation, we infected *miR-15/16*^*fl/fl*^ and *miR-15/16*^*Δ/Δ*^ mice with LCMV. Cycling CD8^+^ and CD4^+^ T cells were measured by 5-ethynyl-2’-deoxyuridine (EdU) incorporation 8 days p.i. Compared to *miR-15/16*^*fl/fl*^ mice, *miR-15/16*^*Δ/Δ*^ mice had significantly more proliferating CD8^+^ and CD4^+^ T cells (Figures 3C and 3D). Additionally, *miR-15/16*^*Δ/Δ*^ mice had increased frequencies and absolute numbers of cells specific for LCMV immunodominant epitopes (GP33 and GP276) both at the peak of the response and long after viral clearance (Figures 3E and 3F). Accumulation of antigen-specific cells in *miR-15/16*^*Δ/Δ*^ mice was cell intrinsic, as it also occurred in mixed bone marrow chimeric mice (Figure 3G). T cells lacking either miR-15a/16–1 or miR-15b/16–2 alone did not exhibit increased proliferation or accumulation of LCMV-specific T cells (Figures S3A–S3D).

These experiments demonstrate that both miR-15/16 clusters act to restrict the accumulation of antigen-specific T cells in response to LCMV infection and suggest that at least part of this effect can be accounted for by decreased proliferation. miR-15/16 effects on the accumulation of CD4^+^ and CD8^+^ T cells are likely mediated by direct binding and posttranscriptional regulation of a network of cell-cycle-associated genes.

### miR-15/16 Restrict the Formation of CD8^+^ Memory Cells

**Figure 4.**
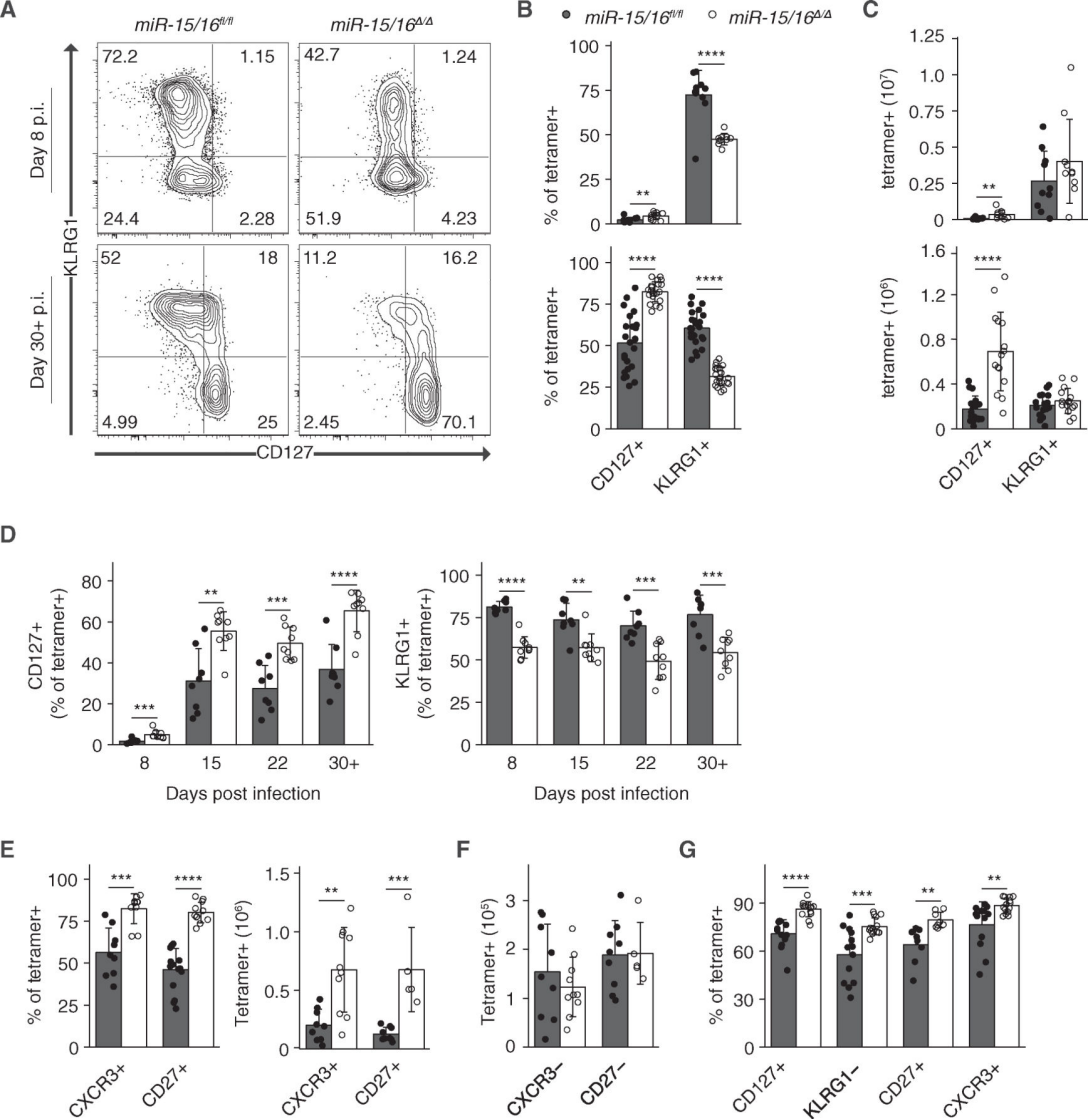
miR-15/16 Restrict the Formation of CD8^+^ Memory Cells (A) Flow cytometry of tetramer^+^ CD8^+^ T cells from spleens harvested 8 days p.i. (top) or 30+ days p.i. (bottom) with LCMV. (B) Quantification of frequencies of tetramer^+^ CD8^+^ T cells from spleens harvested 8 days p.i. (top) or 30+ days p.i. (bottom) with LCMV (n ≥ 10 biological replicates from at least two independent experiments, two-tailed t test). (C) Quantification of absolute numbers of tetramer^+^ CD8^+^ T cells from spleens harvested 8 days p.i. (top) or 30+ days p.i. (bottom) with LCMV (n = 10 biological replicates from two independent experiments, two-tailed t test). (D) Quantification off requencies of tetramer^+^ CD8^+^ T cells from peripheral blood over the course of 30+ days p.i. with LCMV (n ≥ 9 biological replicates from at least two independent experiments, two-tailed t test). (E and F) Quantification of flow cytometry to identify CXCR3^+^ or CD27^+^ (E) and CXCR3^−^ or CD27^−^ (F) populations of LCMV-specific CD8^+^ T cells 30+ days p.i. (n ≥ 5 biological replicates from ≥2 independent experiments, two-tailed t test). (G) Quantification of frequencies of tetramer^+^ CD8^+^ T cells from spleens of mixed bone marrow chimeric mice 30+ days p.i. (n ≥ 8 biological replicates, representative of R3 independent experiments, two-tailed t test). **p < 0.01; ***p < 0.001; ****p < 0.0001. See also Figures S4 and S5.

We hypothesized that the increased accumulation of antigen-specific T cells in LCMV-infected *miR-15/16*^*Δ/Δ*^ mice would be accounted for by increased expansion and survival of virus-specific effector T cells. Surprisingly, however, the absolute number of antigen-specific KLRG1^+^ effector cells was unchanged in *miR-15/16*^*Δ/Δ*^ mice (Figures 4A–4C). Instead, the percentage and absolute number of KLRG1^−^ and CD127^+^ memory cells increased by more than 50%. This effect was apparent as early as 8 days p.i. and stabilized by 15 days p.i. (Figure 4D). Both miR-15/16 clusters contributed to this effect, since single conditional knockout animals displayed only modest changes in these populations (Figures S4A and S4B).

Responding T cell subsets can be distinguished using a variety of markers (Baars et al., 2005; Hikono et al., 2007; Hu et al., 2011; Kaech et al., 2002; Olson et al., 2013; Wherry et al., 2003). *miR-15/16*^*Δ/Δ*^ mice generated higher frequencies and absolute numbers of antigen-specific CD8^+^ T cells with long-lived memory surface phenotypes, including high expression of CD127, CXCR3 and CD27, and low or absent KLRG1 (Figures 4A–4E). Furthermore, even among CD27^−^ and CXCR3^−^ populations, miR-15/16-deficient T cells were enriched for an increased expression of long-lived memory surface marker CD127 (Figure S5). In contrast, effector cells marked as KLRG1^+^, CD127^−^, CXCR3^−^, or CD27^−^ were present at a reduced frequency but equivalent absolute numbers (Figure 4F). Mixed bone marrow chimera experiments demonstrated that these effects were cell intrinsic (Figure 4G).

miR-15/16 deficiencies preferentially affected memory T cell accumulation. The spleens and inguinal lymph nodes (iLNs) of unchallenged *miR-15/16*^*Δ/Δ*^ mice contained increased numbers of both CD8^+^CD44^hi^ and CD4^+^CD44^hi^ memory T cells (Figures S4C and S4D). These cells were not acutely activated, as the frequencies of CD25^+^ T cells were not increased among CD44^hi^ populations (Figure S4E). Memory cells accounted for most of the accumulated T cells. CD44^lo^CD62L^+^ naive T cell numbers were relatively stable, with differences between the control and *miR-15/16*^*Δ/Δ*^ mice reaching significance only for CD8^+^ cells in the spleen (Figure S4F). Taken together, these findings suggest that miR-15/16 specifically restrict the accumulation of long-lived memory cells in unchallenged animals and in response to infection with LCMV, but not at the expense of the naive or effector cell compartment.

### miR-15/16 Restrict Memory CD8^+^ T Cell Differentiation

**Figure 5.**
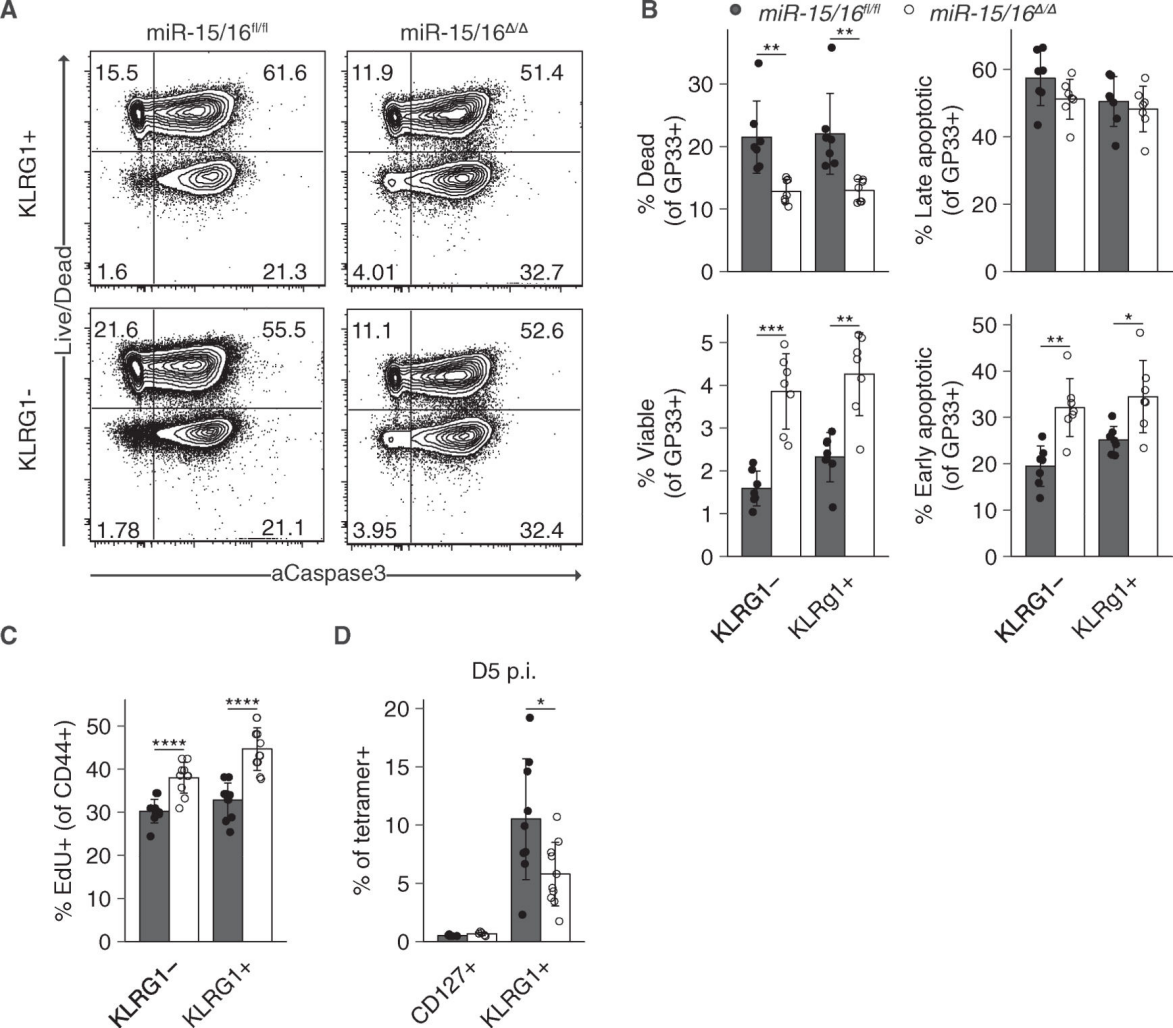
miR-15/16 Restrict Memory CD8^+^ T Cell Differentiation (A) Flow cytometry of GP33^+^ CD8^+^ T cells from spleens harvested 8 days p.i. with LCMV cultured overnight *in vitro* (n = 7 biological replicates from two Independent experiments). (B) Quantification of frequencies of Live/Dead^−^ activated caspase-3^−^ GP33^+^ CD8^+^ T cells from spleens harvested 8 days p.i. with LCMV cultured overnight *in vitro* (n = 7 biological replicates from two independent experiments, two-tailed t test). (C) Quantification of frequencies of EdU^+^ CD44^+^ CD8^+^ T cells from spleens harvested 8 days p.i. with LCMV (n = 7 biological replicates from two independent experiments, two-tailed t test). (D) Quantification of frequencies of MP and TE populations among tetramer^+^ CD8^+^ T cells from spleens harvested 5 days p.i. with LCMV (n = 8 biological replicates from two independent experiments). *p < 0.05; **p < 0.01; ***p < 0.001. See also Figure S6.

We investigated the possibility that memory CD8^+^ T cells preferentially accumulate in miR-15/16-deficient mice due to MP-specific effects on cell survival or proliferation. Following antigen clearance, LCMV-specific TE CD8^+^ T cells undergo a massive contraction phase, leaving behind a population of memory cells that provide protection from future encounters with the same virus. One possible explanation for the increase in MP CD8^+^ T cells in miR-15/16-deficient animals is that they may have a selective survival advantage over miR-15/16-deficient TE cells. To determine whether *miR-15/16*^*Δ/Δ*^ CD8^+^ T cells were exhibiting an MP-specific survival advantage compared to miR-15/16-sufficient cells, splenocytes from mice 8 days p.i. with LCMV were cultured *ex vivo* overnight in the absence of exogenous cytokines. These conditions revealed a significant increase in viable and early apoptotic cells, with a corresponding decrease in dead cells among miR-15/16-deficient, virus-specific CD8^+^ T cells within both MP and TE populations (Figures 5A and 5B). The cell cycle effects of miR-15/16 were also apparent in both MP and TE cells, as measured by *in vivo* EdU incorporation 8 days p.i. with LCMV. Either miR-15b/16–2 or miR-15a/16–1 was sufficient to restrict cell survival and the proliferation of both MP and TE populations (Figures S6A and S6B). *miR-15/16*^*Δ/Δ*^ CD8^+^ T cells were less likely to express the TE marker KLRG1 as early as 5 days p.i. with LCMV (Figure 5D). These results support a model wherein miR-15/16 preferentially restrict memory CD8^+^ T cell accumulation through effects on differentiation. Reciprocal reduction of the alternate effector T cell differentiation is likely balanced by increased survival and proliferation.

### miR-15/16^Δ/Δ^ Memory CD8^+^ T Cells Exhibit Functional Hallmarks of Long-Lived Memory Cells

**Figure 6.**
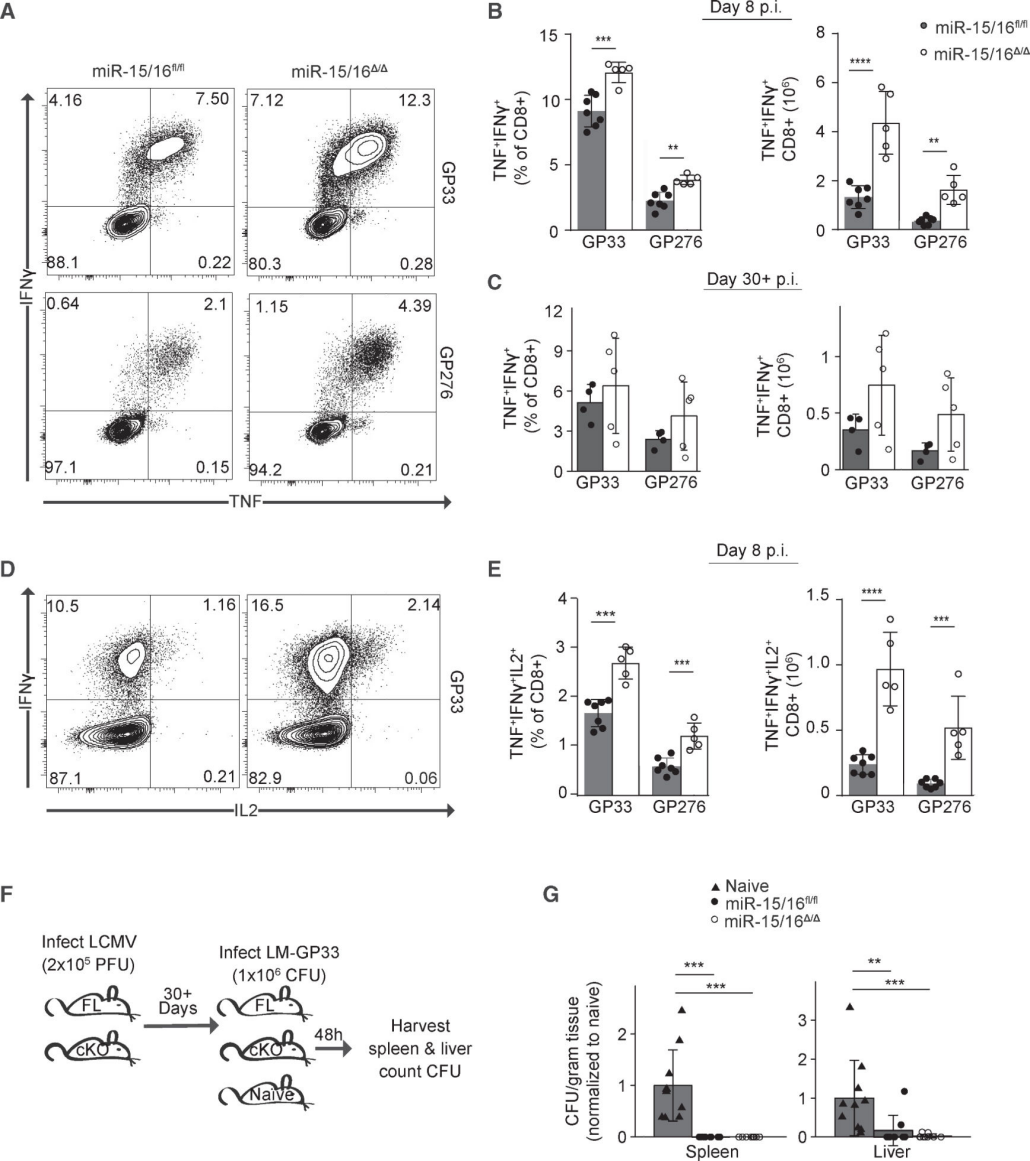
*miR-15/16*^*Δ/Δ*^ Memory CD8^+^ T Cells Exhibit Functional Hallmarks of Long-Lived Memory Cells (A) Flow cytometry of CD8^+^ T cells 8 days p.i. with LCMV responding to splenocytes pulsed with either GP33 (top) or GP276 (bottom). (B) Quantification of cytokine-producing CD8^+^ T cells re-stimulated *in vitro* with peptide-pulsed splenocytes 8 days p.i. (n = 5–7 biological replicates representative of at least two independent experiments, two-tailed t test). (C) Quantification of cytokine-producing CD8^+^ T cells re-stimulated *in vitro* with peptide-pulsed splenocytes 30+ days p.i. (n = 5 biological replicates representative of at least two independent experiments, two-tailed t test). (D) Flow cytometry of CD8^+^ T cells 8 days p.i. with LCMV responding to splenocytes pulsed GP33 and analyzed for multi-functional cytokine production. (E) Quantification of multi-functional cytokine-producing CD8+ T cells re-stimulated *in vitro* with peptide-pulsed splenocytes 8 days p.i. (n = 5–7 biological replicates from a single experiment, two-tailed t test). (F) Experimental outline of direct re-challenge model. (G) LM-GP33 colony forming units (c.f.u.) from the spleen and liver of mice infected with LM-GP33 30+ days p.i. with LCMV and harvested 48 h later normalized to the naive group of each respective experiment (n ≥ 9 biological replicates from two independent experiments, Mann-Whitney U test). *p < 0.05; **p < 0.01; ***p < 0.001; ****p < 0.0001.

We further characterized the quality of *miR-15/16*^*Δ/Δ*^ memory CD8^+^ T cell responses to an antigen re-challenge and secondary infection. First, we pulsed splenocytes from LCMV-infected mice with GP33 or GP276 *ex vivo.* Consistent with the increases in antigen-specific cells detected by tetramer staining, greater frequencies and absolute numbers of CD8^+^ T cells from *miR-15/16*^*Δ/Δ*^ mice produced the effector cytokine’s tumor necrosis factor (TNF) and IFNγ 8 days p.i. (Figures 6A and 6B), with a non-significant trend toward an increase at day 30+ p.i. (Figure 6C). Furthermore, greater frequencies and absolute numbers of CD8^+^ T cells from *miR-15/16*^*Δ/Δ*^ mice exhibited a polyfunctional phenotype, characterized by production of TNF, IFNγ, and IL2 8 days p.i. (Figures 6D and 6E).

To assess the effectiveness of miR-15/16-deficient antigen-specific memory CD8^+^ T cells *in vivo,* we re-challenged animals 30 days p.i. with LCMV with *Listeria monocytogenes* expressing GP33 (LM-GP33) (Figure 6F). CD27^low^ memory CD8^+^ T cells provide superior protection to *Listeria,* compared to CD27^+^ cells (Olson et al., 2013). *miR-15/16*^*Δ/Δ*^ and *miR-15/16*^*fl/fl*^ animals had equivalent numbers of this subset of memory T cells (Figure 4F) and exerted equivalent control of the LM-GP33 bacterial burden (Figure 6G). Thus, miR-15/16 are dispensable for the establishment of functionally protective CD8^+^ memory T cells, but is required for the generation of a normally proportioned memory cell pool. In other contexts, the imbalanced increase in CD27^+^ T cells in *miR-15/16*^*Δ/Δ*^ animals may generate improved protection or hyperactive secondary immune responses (Hikono et al., 2007; Roberts et al., 2010).

### A Network of Memory-Associated Genes Is Regulated by miR-15/16

**Figure 7.**
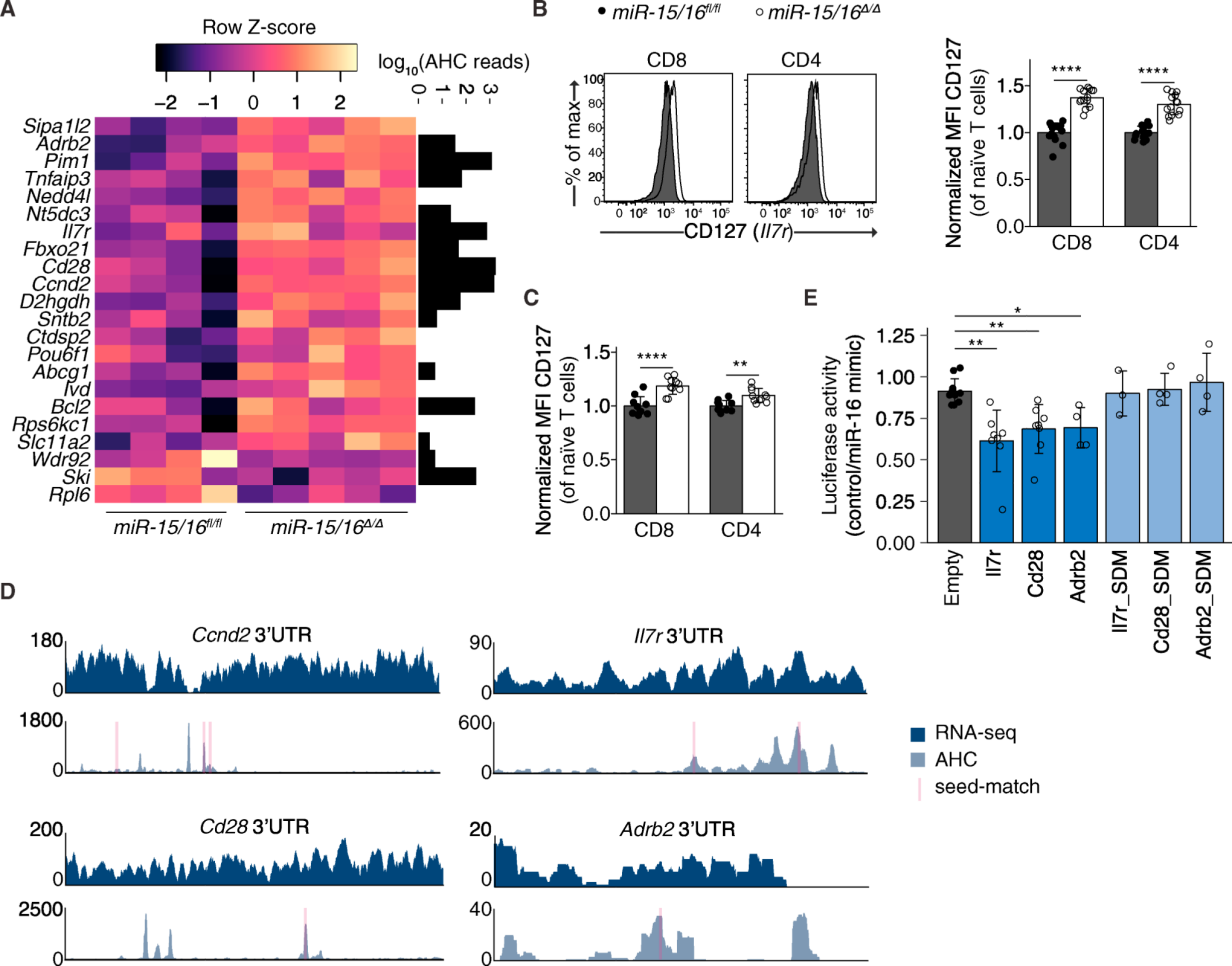
A Network of miR-15/16 Targets Is Upregulated in Memory Cells (A) Row z-score normalized expression of genes found to be upregulated in memory cells compared to day 8 p.i. effector cells, differentially expressed in *miR-15/16*^*Δ/Δ*^ cells (p value ≤ 0.1), and containing at least one miR-15/16 seed match in their 3′UTR. Black bars indicate AHC reads at miR-15/16 seed matches. (B) Flow cytometry of CD127 on naive CD4^+^ and CD8^+^ T cells (n ≥ 14 biological replicates from three independent experiments, two-tailed t test). (C) Flow cytometry of CD127 on naive CD4^+^ and CD8^+^ T cells from mixed bone marrow chimeric mice (n ≥ 9 biological replicates from two independent experiments, two-tailed t test). (D) RNA-seq (top) and AHC (bottom) for the 3′UTRs of *Ccnd2, Il7r, Cd28,* and *Adrb2* with red-shaded regions indicating the locations of miR-15/16 seed matches. (E) Relative luciferase activity between empty vector, *Il7r* 3′UTR, *Cd28* 3′UTR, *Adrb2* 3′UTR, and all three 3′UTRs with their respective miR-15/16 seed matches scrambled (n R 3 biological replicates from at least two independent experiments, two-tailed t test). *p < 0.05; **p < 0.01; ***p < 0.001; ****p < 0.0001. See also Figure S7.

In order to shed light on the mechanistic targets of miR-15/16 that contribute to memory CD8^+^ T cell differentiation, we coupled our RNA-seq and AHC data with existing data that defined a set of genes upregulated in memory T cells following LCMV infection (Kaech et al., 2002). Since long-lived memory CD4^+^ and CD8^+^ T cells share more than 95% similarity in gene expression (Chang et al., 2014), we reasoned that experiments conducted using CD4^+^ T cells could provide valuable insights into miR-15/16 regulation of CD8^+^ T cells. Twenty-two memory-associated genes contained a 3′UTR seed match and were differentially expressed in *miR-15/16*^*Δ/Δ*^ T cells (p ≤ 0.1). Of these, 19 were upregulated in *miR-15/16*^*Δ/Δ*^ cells (Figure 7A, left). AHC detected AGO2 binding at miR-15/16 seed-match sites in most of these genes, further supporting direct regulation by miR-15/16 (Figure 7A, right). Among the genes upregulated in *miR-15/16*^*Δ/Δ*^ T cells and containing AGO2 binding at miR-15/16 seed-match sites, *Adrb2* (Slota et al., 2015), *Pim1* (Knudson et al., 2017), *Il7r* (Nanjappa et al., 2008), *Cd28,* and *Bcl2* (Kurtulus et al., 2011) have been extensively studied in the context of CD8^+^ memory.

This approach identified numerous putative targets of miR-15/16 that may contribute to memory cell differentiation. *Il7r* was of particular interest, as it has been previously reported that the addition of exogenous recombinant IL-7 can boost memory formation (Nanjappa et al., 2008). Surface CD127 protein abundance was elevated in naive *miR-15/16*^*Δ/Δ*^ CD8^+^ and CD4^+^ T cells (Figure 7B). This effect was cell intrinsic, as it was also observed in mixed bone marrow chimeric mice (Figure 7C). Similar to the well-characterized target of miR-15/16, *Ccnd2,* the *Il7r* 3′UTR contains multiple major AHC peaks corresponding to miR-15/16 seed matches (Figure 7D). Dual luciferase assays in *miR-15/16*^*Δ/Δ*^ cells transfected with control (CM) or miR-16 mimic validated the capacity of miR-16 to directly target the *Il7r* 3′UTR (Figure 7E). Site-directed mutagenesis of both miR-15/16 seed-match sites completely rescued luciferase expression to CM-treated levels, proving that the effects of miR-15/16 on the *Il7r* 3′UTR are dependent upon these seed matches (Figure 7E). These results provide strong evidence that *Il7r* is a bona fide target of miR-15/16.

AHC peaks also sharply coincided with miR-15/16 3′UTR seed matches in many of these putative memory-associated targets (Figure S7), especially *Cd28* and *Adrb2* (Figure 7D). Dual luciferase assays confirmed these two additional targets; mutating the miR-15/16 seed matches within their 3′UTRs completely rescued luciferase expression to CM-treated levels (Figure 7E). While it is unlikely that any one of these targets could account for the effects of miR-15/16 on CD8^+^ memory cell differentiation, regulation of this network of memory-associated genes provides a plausible mechanism by which miR-15/16 may regulate memory differentiation.

## DISCUSSION

The experiments in this study demonstrated roles for miR-15/16 in the restriction of T cell cycle, survival, and CD8^+^ memory cell differentiation through the direct targeting of networks of genes associated with cell cycle, signaling, metabolism, and survival. Both the miR-15a/16–1 and miR-15b/16–2 clusters were sufficient to restrict cell cycle, survival, and CD8^+^ memory cell differentiation during the response to LCMV infection. Moreover, all four mature miRNAs from these clusters are downregulated during the response, and both miR-15b and miR-16 remain low long after viral clearance. Given the importance of the quantity and quality of cells generated in response to an immunologic insult such as infection or cancer, our findings mark miR-15/16 as critical tuners of the cell-mediated adaptive immune response.

miRNAs exert their biological effects through multiple target genes (Pua et al., 2016), and our data indicate that miR-15/16 regulate a remarkably extensive target network. We combined biochemical and bioinformatic approaches to map AGO2 binding at thousands of miR-15/16 seed-match sequences in mRNAs and linked this with gene expression analysis in *miR-15/16*^*Δ/Δ*^ T cells to identify a large network of high-confidence direct miR-15/16 target genes that restrict cell cycle and CD8^+^ long-lived memory T cell differentiation. This approach is best suited to highly expressed miRNAs like miR-15/16 since seed-match occupancy correlated robustly with miRNA AGO2 occupancy. Deletion of both the endogenous *Mirc30* (*miR-15a/16–1*) and *Mirc10* (*miR-15b/16–2*) clusters was also critical to unveiling this large set of target genes.

At the peak of infection with LCMV, antigen-specific CD8^+^ T cells divide as rapidly as once every 2 h (Yoon et al., 2010) and complete >14 divisions in 7 days p.i. (Blattman et al., 2002). Even with this remarkably rapid rate of division, removal of miR-15/16 increased the accumulation of virus-specific cells, emphasizing the degree of control exerted by miR-15/16. Previous studies in CLL as well as malignant pleural mesothelioma demonstrated that miR-15/16 are frequently downregulated or deleted and directly target several cell cycle genes (Klein et al., 2010; Reid et al., 2013). Our findings expand this cell cycle target network and extend the influence of miR-15/16 to the control of antigen-driven T cell clonal selection.

miR-150, which is also downregulated in response to T cell activation (Figures 1A and 1B), also restricts memory cell development (Ban et al., 2017; Chen et al., 2017). However, unlike miR-150, miR-15/16 expression is maintained at reduced levels during the memory phase (Figure 1B), suggesting that targets of miR-15/16 may be critical to the maintenance of memory cells. In fact, a number of miR-15/16 targets, including *Bcl2* (Kurtulus et al., 2011), *Pim1* (Knudson et al., 2017), *Il7r* (Kaech et al.,2003), and *Cd28* (Klein Geltink et al., 2017), have been linked with the maintenance and functional capacity of the memory CD8^+^ T cell pool. Interestingly, *Il7r*/CD127 may represent not only a target of miR-15/16, but also part of a feed-forward negative regulator of miR-15/16 expression through STAT5, which is activated downstream of CD127 signaling, and a direct transcriptional repressor of *miR-15b/16–2* (Li et al., 2010; Lindner et al., 2017). This feed-forward circuit may play important roles in other lymphocyte cell fate decisions. For example, miR-15 family activity was inversely correlated with IL-7 receptor surface expression in pre-B cells (Lindner et al., 2017).

We conclude that miR-15/16 represents an important node in the coordinate regulation of cell proliferation, survival, and early differentiation of memory CD8^+^ T cells. High precursor frequencies of long-lived memory cells reduce the proliferative burden required for a response to secondary challenge, thereby preserving mitochondrial integrity and allowing for a more productive secondary response (Sheridan et al., 2014). Therefore, reducing miR-15/16 expression might be beneficial in the context of vaccination and cancer immunotherapies where the capacity of memory CD8^+^ T cells to expand and respond is critical for protection. Future studies investigating the regulation of miR-15/16 in response to T cell stimulation, as well as the maintenance of its suppression in memory CD8^+^ T cells, could identify up-stream drivers of memory formation and maintenance and provide potential therapeutic targets to modulate T cell memory.

## STAR★METHODS

### LEAD CONTACT AND MATERIALS AVAILABILITY

Further information and requests for resources and reagents should be directed to and will be fulfilled by the Lead Contact, K. Mark Ansel (mark.ansel@ucsf.edu). Mirc10^tm1Mtm^ ES cells and mice generated in this study have been deposited to the Mutant Mouse Resource & Research Center (MMRRC:037643-JAX).

### EXPERIMENTAL MODEL AND SUBJECT DETAILS

Mice generated from Mirc10^tm1Mtm^ ES cells (Park et al., 2012)} were crossed with *Rosa26-Flp* mice (GT(ROSA)26Sor^tm1(FLP1)Dym^; 009086, Jackson Laboratory) to delete the selection cassette, yielding a loxP-flanked allele. “*miR-15/16*^*fl/fl*^*”* animals were generated by crossing the resulting *miR-15b/16–2*^*fl/fl*^ animals with *miR-15a/16–1*^*fl/fl*^ (Mirc30^tm1.1Rdf^) animals (Klein et al., 2010). “*miR-15/16*^*Δ/Δ*^*”* animals were generated by further crossing *miR-15/16*^*fl/fl*^ animals with *CD4-Cre* mice (Tg(CD4-cre)1Cwi; 4196, Taconic) (Sawada et al., 1994). For hematopoietic chimeras, B6-Ly5.1/Cr (CD45.1+; Charles River) mice were lethally irradiated (2×550 rad), reconstituted with 5×10^6^ bone marrow cells, and analyzed 8–10 weeks later. Male and female age and sex matched mice were used between 5 to 12 weeks of age. All mice were housed and bred in specific pathogen-free conditions in the Animal Barrier Facility at the University of California, San Francisco. Animal experiments were approved by the Institutional Animal Care and Use Committee of the University of California, San Francisco.

#### LCMV model

Animals were infected intraperitoneally with 2×10^5^ plaque forming units (p.f.u.) LCMV Armstrong. Mice were bled retroorbitally with heparinized 75mm hematocrit tubes (Drummond) or sacrificed for spleen harvesting. Splenocytes were re-stimulated *in vitro* with 0.2μg/ml GP33–41 (KAVYNFATM) or GP276–286 (SGVENPGGYCL) for 5 hours in the presence of brefeldin A. For EdU labeling, 1mg EdU was injected retroorbitally 16 hours before sacrifice and detected using the Click-iT EdU flow cytometry assay kit (Thermo Fisher Scientific).

#### Listeria monocytogenes model

Animals 30+ days post-infection (p.i.) with LCMV Armstrong and naive control mice (no prior infection with LCMV) were infected with 1–2×10^5^ colony forming units (c.f.u.) of LM-GP33. Two days later, spleen and liver samples were harvested, and bacterial content was analyzed as described (Shehata et al., 2018). Spleens and livers were isolated, weighed, placed in 1% FBS in PBS, and homogenized. Single-cell suspensions were made from the spleens and livers of recipient mice in PBS containing 0.5% Triton X-100. Serial dilutions of the supernatants were inoculated on brain-heart-infusion agar plates and were incubated for 24h at 37°C. Bacterial colonies were enumerated, and c.f.u. were normalized per gram of spleen or liver plated. Data were normalized to the mean c.f.u of naive control mice.

### METHOD DETAILS

#### *in Vitro* Cultures and qPCR

CD4^+^ T cells from the spleen and lymph nodes of mice were enriched by Dynabead-positive selection (Invitrogen, L3T4. Cells were stimulated with biotinylated anti-CD3 (clone 2C11, 1μg/ml) and anti-CD28 (clone 37.51, 0.5μg/ml) for 3 days on plates pre-coated with NeutrAvidin (5μg/ml, Thermo Fischer Scientific), then rested with 20 units/mL recombinant IL-2 (National Cancer Institute) for an additional 2 days. Cells were cultured in DMEM high glucose media supplemented with 10% FCS, pyruvate, nonessential amino acids, MEM vitamins, L-arginine, L-asparagine, L-glutamine, folic acid, beta mercaptoethanol, penicillin, and streptomycin. For miRNA qPCR analysis, cells were lysed in Trizol LS (Life Technologies), total RNA isolated, and RNA quantified on an ND1000 Spectrophotometer (NanoDrop). Reverse transcription of miRNA was performed with the NCode miRNA First-Strand cDNA Synthesis Kit (Life Technologies). Forward primers were the mature miRNA sequences (miRbase.org) and a universal reverse primer was used from the kit. Expression values were normalized to the 5.8S ribosomal RNA (F: ATCGTAGGCACCGCTACGCCTGTCTG). qPCR was performed in technical duplicates using FastStart Universal SYBR Green Master mix (Roche) on a Realplex^2^ instrument (Eppendorf).

#### Flow Cytometry

Cells were harvested from thymus, spleen, and lymph nodes by mashing through 70μM filters. Splenic and blood RBCs were lysed with ACK buffer for 5 min at room temperature. Single cell suspensions were prepared in PBS 2% FCS and stained for analysis on an LSRFortessa (Becton Dickenson). Tetramer reagents were generated mixing biotinylated peptide-MHC monomers with fluorophore-conjugated Streptavidin. ForGP33 (H-2Db GP33–41 KAVYNFATM), 200ug peptide-MHC monomer was incubated with 318uLStrep-tavidin-PE (Life Sciences). For GP276 (H-2Db GP276–286 SGVENPGGYCL), 200ug peptide-MHC monomer was incubated with 176uL Streptavidin-APC (Life Sciences). 1/10 total volume of Streptavidin was added every 15 minutes on ice in the dark and gently mixed. For EdU assays, cells were stained with anti-CD3 PE-dazzle 594, anti-CD4 PerCP-Cy5.5, anti-CD8 BV785. Cells were then fixed and permeabilized and labeled with EdU AF647 (Thermo-Fisher) according to the manufacturer’s instructions. For transcription factor stains, cells were fixed and permeabilized using the Foxp3 Transcription Factor Staining Buffer Set (eBioscience). For cytokine stains, cells were fixed for 8 min at room temperature with 4% paraformaldehyde, and permeabilized with 0.5% saponin buffer. Normalized MFI is geometric mean fluorescence intensity normalized to the average for the *miR-15/16*^*fl/fl*^ samples in each independent experiment. Data were analyzed with FlowJo.

#### AGO2 HITS-CLIP

Libraries were constructed as previously described (Loeb et al., 2012) using CD4^+^ T cells, with the following modifications. To eliminate nuclei, SDS was excluded from the lysis buffer and physical lysis was not performed with a needle. SDS was included before adding the lysate to anti-AGO2 coated beads. AGO2 immunoprecipitation was performed using a monoclonal antibody (Wako; clone 2D4). To increase resolution and precision, we used a 10% TBE gel (BioRad) for extracting final PCR products. 10% PEG was added to 3′ and 5′ linker ligation steps to improve ligation efficiency. A randomized dinucleotide was added to the 3′ linker to reduce ligation bias. Maximum read depths across mature miRNAs and miRNA targets were generated using the samtools package (Li et al., 2009). Bedfiles used for mapping to transcriptomic regions were acquired from the UCSC table browser using the mm10 reference genome.

#### RNA sequencing

1×10^6^ CD4^+^ T cells cultured *as described above* were lysed in Trizol (Life Technologies) and RNA isolated using miRNeasy Micro Kit (QIAGEN) with on-column DNase digestion. cDNA was synthesized using the TruSeq PolyA library kit. Single-end 50 base-pair RNA sequencing was performed on the Illumina HiSeq 4000. Alignment was performed using STAR_2.4.2a (Dobin et al., 2013) against the Ensembl Human GRCm38.78 alignment genome. Differential expression was tested using DESeq2 v1.14.0 (Love et al., 2014). Analysis was performed on datasets filtered on genes with an average of at least 5 reads-per-million across all samples. Expression values were analyzed as log2(fold change) of miR-15/16 deficient T cells compared with miR-15/16 sufficient T cells. Raw data and processed datasets have been deposited in GEO.

The cumulative distribution function plot was generated in R (www.r-project.org) to display the log2(*miR-15/16*^*Δ/Δ*^*/miR-15/16*^*fl/fl*^) against the cumulative distribution of genes. The expression values from all genes lacking a miR-15/16 3′UTR seed match were plotted along with all genes predicted to be targeted by miR-15/16 by TargetScan (www.targetscan.org), all genes containing a miR-15/16 3′UTR seed match, and the subset of all genes containing a miR-15/16 3′UTR seed match with above 5 AHC reads mapping to the seed match. Gene Set Enrichment Analysis (http://software.broadinstitute.org/gsea/index.jsp) was performed using the set of genes with an average of at least 5 reads-per-million across all samples. Plotted were the enrichment curve and rank order location of each gene contained within the KEGG Cell Cycle gene set from most upregulated to most downregulated (left to right).

#### Luciferase Assays

CD4^+^ T cells were transfected on day 1 of culture with luciferase reporter constructs and/or 500nM miRIDIAN miRNA mimics (Dharmacon) using the Neon Transfection System (Thermo Fisher Scientific). Luciferase activity was measured 24 h after transfection with the Dual Luciferase Reporter Assay System (Promega) and a FLUOstar Optima plate-reader (BMG Labtech). The near full-length 3′UTRs of *Il7r, Cd28, and Adrb2* were cloned into the psiCHECK-2 luciferase reporter construct (Promega). Primers for cloning and site-directed-mutagenesis (SDM) were: *Il7r* F: CAGGCTCGAGATTATAAGAAAACCCTTCCATCGACAACC, *Il7r* R: GATGCGGCCGCTTCCAGAAA ATAGCGCATGCTTGTATTTG; *Cd28* F: TAGCTCGAGCAGGGACCCCTATCCAGAAG, *Cd28* R: CTAGCGGCCGCAGTCAATGAAT AAATATTTATTGCAGGCTAAGC; *Adrb2* F: CAGCTCGAGAGGCTTTCTACTCTCTAAGACCC, *Adrb2* R: CAGGCGGCCGCCCAC TCATCGGTCACGACAC. SDM was performed using QuickChange Lightning Site-Directed Mutagenesis Kit (Agilent) using primers: *Il7r* SDM F: GATAACCACAACAGTCCTGAATGCTTGATTATATTCTCAGG, *Il7r* SDM R: CCTGAGAATATAATCAAGCATTCAGGA CTGTTGTGGTTATC; *Cd28* SDM F: GAAAACTATGTCACTTGTCCTGATTATTGTAAGAGTCTAAGAAC, *Cd28* SDM R: GAAAACTATG TCACTTGTCCTGATTATTGTAAGAGTCTAAGAAC; *Adrb2* SDM F: GAATGATATATATTGTCCTGGGAAATCCATATCTAAAGGAG AGAG, *Adrb2* SDM R: CTCTCTCCTTTAGATATGGATTTCCCAGGACAATATATATCATTC.

### QUANTIFICATION AND STATISTICAL ANALYSIS

Excel (Windows) and R were used for data analysis. Some plots were generated using the Bioconductor package, plotGrouper (Gagnon, 2018). For all figures, bar graphs display mean ± s.d. and each point represents an individual mouse unless otherwise stated. *p < 0.05, **p < 0.01, ***p < 0.001, and ****p < 0.0001 for significance. Parametric statistics were employed except in cases of non-normally distributed data. In those cases, differences between groups were assessed using a Mann-Whitney U test.

### DATA AND CODE AVAILABILITY

The RNA-seq data reported in this paper are archived at the NCBI Gene Expression Omnibus database (accession number GEO: GSE111568 and GEO: GSE130655).

## Supplementary Material

1

2

## Figures and Tables

**KEY RESOURCES TABLE:** 

REAGENT or RESOURCE Antibodies	SOURCE	IDENTIFIER
Anti-CD3 PE-dazzle 594 (145-2C11)	BioLegend	Cat# 100348; RRID:AB_2564029
Anti-CD8a BV785 (53-6.7)	BioLegend	Cat# 100749; RRID:AB_11218801
Anti-CD8a BUV805 (53-6.7)	BD Biosciences	Cat# 564920; RRID:AB_2716856
Anti-CD4 PerCP-Cy5.5 (RM4-5)	Thermo Fisher Scientific	Cat# 45-0042-80; RRID:AB_906231
Anti-Foxp3 APC (FJK-16 s)	Thermo Fisher Scientific	Cat# 17-5773-82; RRID:AB_469457
Anti-CXCR3 FITC (CXCR3-173)	Thermo Fisher Scientific	Cat# 11-1831-82; RRID:AB_11040010
Anti-CD127 BV421 (A7R34)	BioLegend	Cat# 135024; RRID:AB_11218800
Anti-CD62L BV605 (MEL-14)	BioLegend	Cat# 104438; RRID:AB_2563058
Anti-CD45.1 BV785 (A20)	BioLegend	Cat# 110743; RRID:AB_2563379
Anti-CD45.2 BUV395 (104)	BD Biosciences	Cat# 564616; RRID:AB_2738867
Anti-CD43 PerCP-Cy5.5 (1B11)	BioLegend	Cat# 121223; RRID:AB_893336
Anti-KLRG1 PE-Cy7 (2F1/KLRG1)	BioLegend	Cat# 138416; RRID:AB_2561736)
Anti-CD44 Af700 (IM7)	Thermo Fisher Scientific	Cat# 56-0441-82; RRID:AB_494011
Anti-CD27 FITC (LG.7F9)	Thermo Fisher Scientific	Cat# 11-0271-82; RRID:AB_465001
Anti-GR1 APC-Cy7 (RB6-8C5)	BioLegend	Cat# 108423; RRID:AB_2137486
Anti-CD11b APC-Cy7 (M1/70)	BD Biosciences	Cat# 557657; RRID:AB_396772
Anti-active caspase 3 (C92-605)	BD Biosciences	Cat# 559565; RRID:AB_397274
Anti-cd19 APC-H7 (1D3)	BD Biosciences	Cat# 560245; RRID:AB_1645233
Anti-TNF AF700 (MP6-XT22)	BD Biosciences	Cat# 558000; RRID:AB_396980
Anti-IFNγ FITC (XMG1.2)	BioLegend	Cat# 505806; RRID:AB_315400
Anti-IL2 APC (JES6-5H4)	Thermo Fisher Scientific	Cat# 17-7021-81; RRID:AB_469489
Anti-CD16/32 (2.4G2)	Bio X Cell	Cat# BE0307; RRID:AB_2736987
Anti-Rabbit A647 (Donkey polyclonal)	BioLegend	Cat# 406414; RRID:AB_2563202
Anti-CD3 (2C11)	Bio X Cell	Cat# BP0001-1; RRID:AB_1107634
Anti-CD28 (37.51)	Bio X Cell	Cat# BE0015-1; RRID:AB_1107624
Anti-AGO2 (2D4)	Wako	Cat# 018-22021; RRID:AB_1106838
Bacterial and Virus Strains		
LCMV (Armstrong)	in house, M. Matloubian lab	NA
Listeria monocytogenes	in house, S. Sanjabi lab	NA
Chemicals, Peptides, and Recombinant Proteins		
GP33	NIH	NA
GP276	NIH	NA
Streptavidin PE	Life Sciences	Cat# S-866
Streptavidin APC	Life Sciences	Cat# S-868
Recombinant IL-2	NCI	NA
Critical Commercial Assays		
Click-iT EdU Alexa Fluor 647 Flow Cytometry Assay Kit	Thermo-Fisher	Cat# C10419
Foxp3/Transcription Factor Staining Buffer Set	eBioscience	Cat# 00-5523-00
Deposited Data		
RNA sequencing	This study	GEO: GSE111568
Ago2 HITS-CLIP	This study	GEO: GSE130655
Experimental Models: Organisms/Strains		
Cd4-cre Transgenic (B6.Cg-Tg(CD4-cre)1Cwi	Taconic	Cd4-cre Transgenic (Model #: 4196)
miR15a/16-1 fl Mirc30^tm11Rdf^	The Jackson Laboratory	JAX: 017641
miR15b/16-2 fl Mirc10^tm1Mtm^	This study; now available via The Jackson Laboratory	MMRRC Stock No: 37643-JAX
NCI B6-Ly5.1/Cr (B6.SJL-Ptprc^a^Pepc^b^/BoyCrCrl)	Charles River	NCI B6-Ly5.1/Cr (Strain code: 564)
Oligonucleotides		
miR15a-5p miRIDIAN miRNA mimic	Dharmacon	C-310510-05-0002
miR16 miRIDIAN miRNA mimic	Dharmacon	C-310511-05-0002
miRIDIAN control mimic	Dharmacon	CN-001000-01-05
miR15b-5p miRIDIAN miRNA mimic	Dharmacon	C-310378-05-0002
Recombinant DNA		
Plasmid: psiCheck-2	Promega	C8021
Plasmid: psiCHECK-2-IL7r	In this study	NA
Plasmid: psiCHECK-2-Cd28	In this study	NA
Plasmid: siCHECK-2-Adrb2	In this study	NA
Software and Algorithms		
Flow jo V9	FlowJo, LLC	NA
Plot Grouper	Bioconductor	https://doi.org/10.18129/B9.bioc.plotGrouper
microRNA.TEvMP	This study, available at GitHub	https://github.com/jdgagnon/microRNA.TEvMP
DE.AHC	This study, available at GitHub	https://github.com/jdgagnon/DE.AHC

## Highlights

- miR-15/16 and their target mRNAs are abundant in Ago2 complexes in T cells
- miR-15/16 regulate the number and type of T cells generated during viral infection
- miR-15/16 target acts in pathways critical for T cell expansion and differentiation
