# Supplementary material for: miR-15/16 Restrain Memory T Cell Differentiation, Cell Cycle, and Survival
Source: Cell Rep. Author manuscript; Available in PMC 2019 Aug 29. (PMC6715152; doi:10.1016/j.celrep.2019.07.064)
Supplement: 1 [file NIHMS1538157-supplement-1.pdf]

Cell Reports, Volume *28*

Supplemental Information

miR-15/16 Restrain Memory T Cell Differentiation, Cell Cycle, and Survival

John D. Gagnon, Robin Kageyama, Hesham M. Shehata, Marlys S. Fassett, Darryl J. Mar, Eric J. Wigton, Kristina Johansson, Adam J. Litterman, Pamela Odorizzi, Dimitre Simeonov, Brian J. Laidlaw, Marisella Panduro, Sana Patel, Lukas T. Jeker, Margaret E. Feeney, Michael T. McManus, Alexander Marson, Mehrdad Matloubian, Shomyseh Sanjabi, and K. Mark Ansel

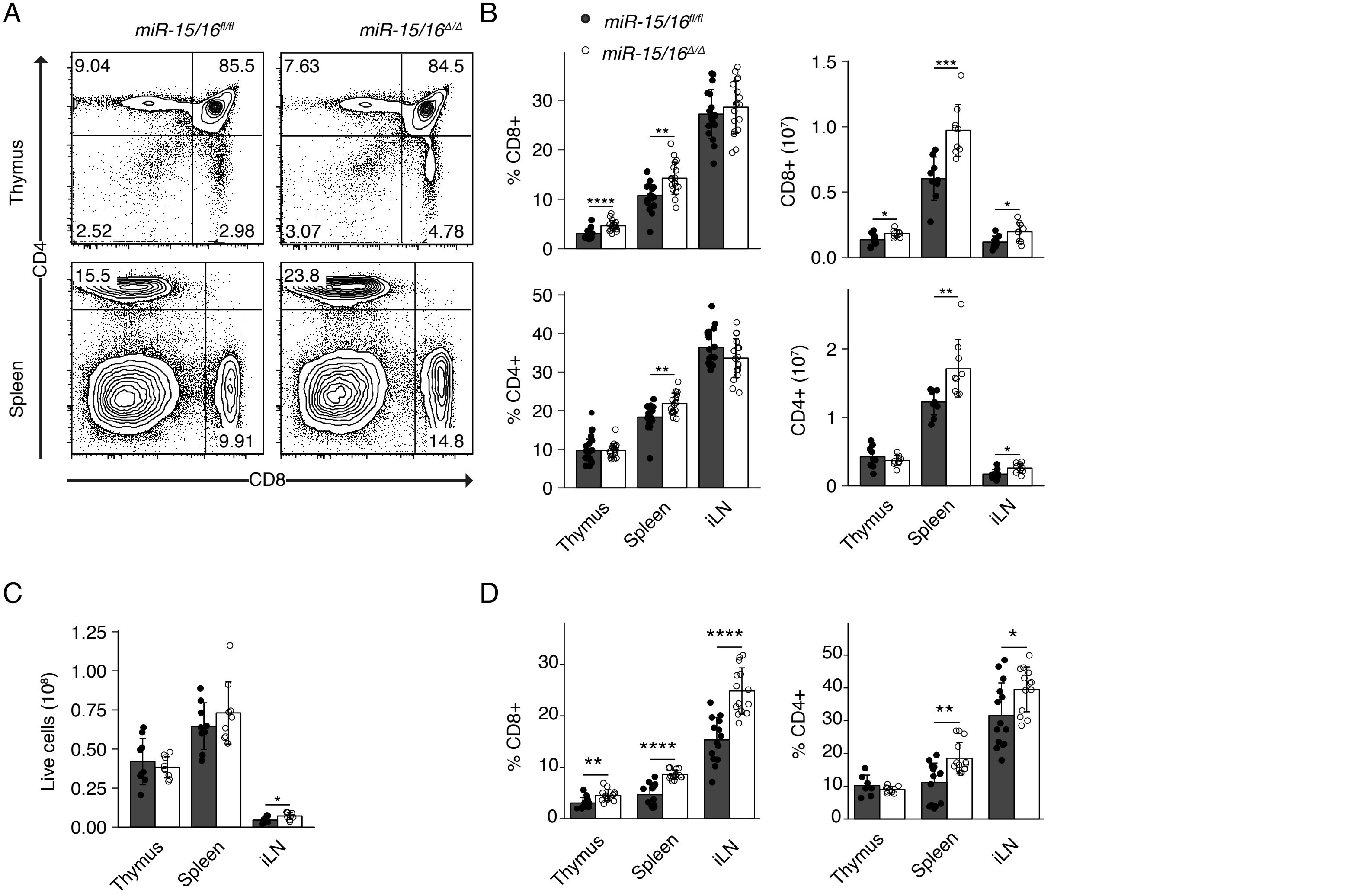

Figure S1. miR-15/16 restrict CD4$^+$ and CD8$^+$ T cell accumulation in unchallenged animals, Related to Figure 1.

(A) Flow cytometry of CD4$^+$ and CD8$^+$ T cells in primary and secondary lymphoid tissues collected from miR-15/16$^{fl/fl}$ and miR-15/16$^{\Delta/\Delta}$ mice. (B) Quantification of frequencies and absolute numbers of CD4$^+$ and CD8$^+$ T cells in primary and secondary lymphoid tissues collected from miR-15/16$^{fl/fl}$ and miR-15/16$^{\Delta/\Delta}$ mice (n = 10 biological replicates from 2 independent experiments, two-tailed t test). (C) Quantification of absolute numbers of live cells within primary and secondary lymphoid tissues collected from miR-15/16$^{fl/fl}$ and miR-15/16$^{\Delta/\Delta}$ mice (n ≥ 9 biological replicates from 3 independent experiments, two-tailed t test). (D) Quantification of frequencies of CD4$^+$ and CD8$^+$ T cells in primary and secondary lymphoid tissues collected from miR-15/16$^{fl/fl}$ and miR-15/16$^{\Delta/\Delta}$ mixed bone marrow chimeric mice (n ≥ 10 biological replicates from 3 independent experiments, two-tailed t test). *, P < 0.05; **, P < 0.01; ***, P < 0.001; ****, P < 0.0001.

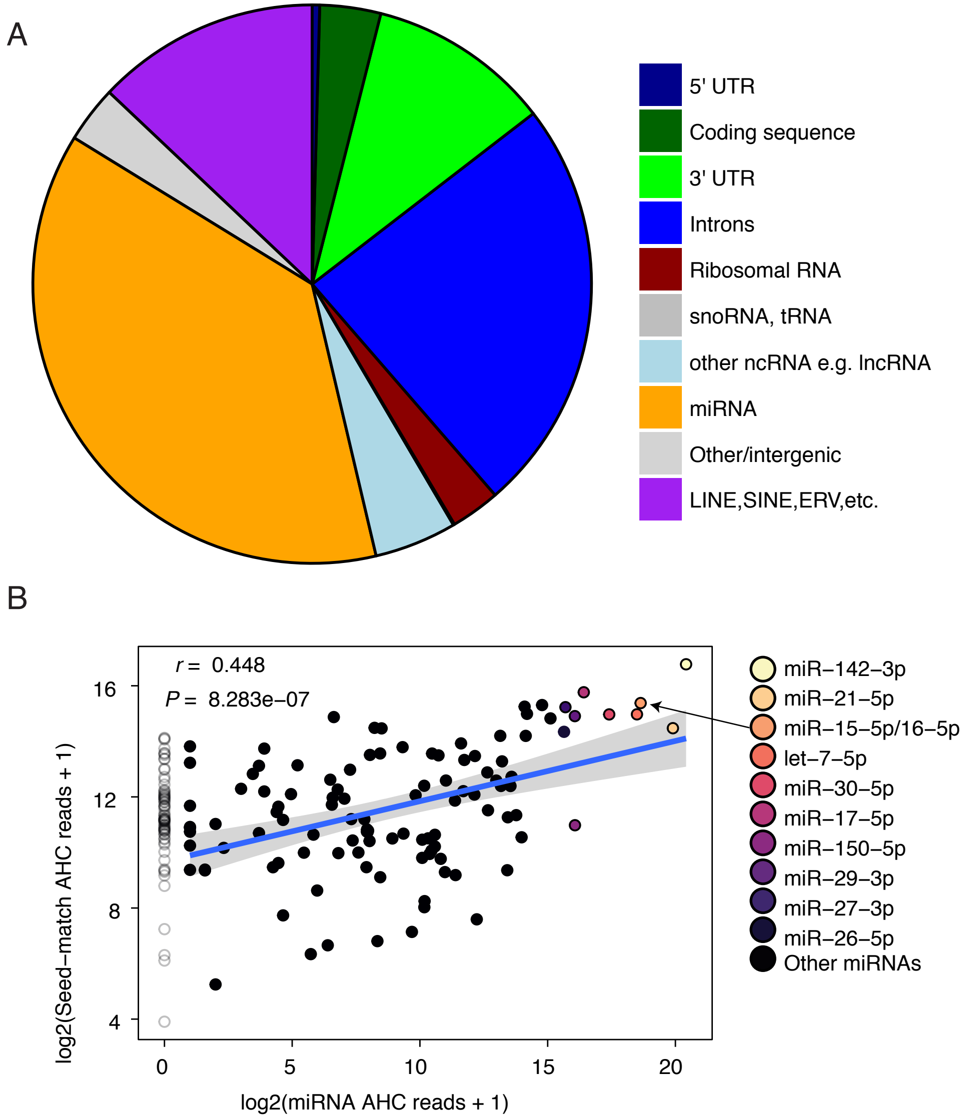

Figure S2. miR-15/16 bind and regulate a large network of direct target RNAs in T cells, Related to Figure 2.

(A) Fraction of AHC reads mapping to genomic loci. (B) Correlation between AHC reads mapping to mature miRNAs and their respective TargetScan predicted seed-matches.

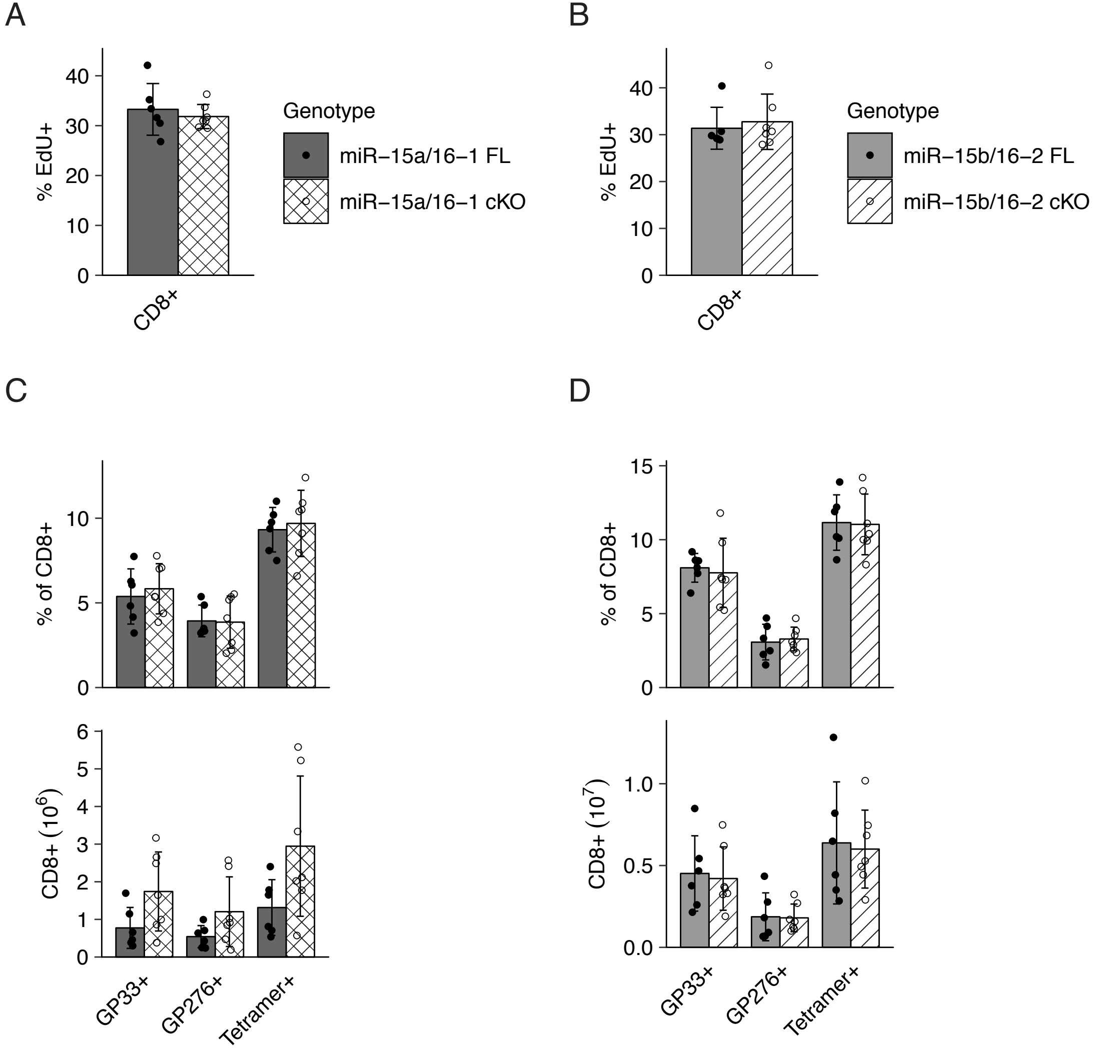

Figure S3. miR-15a/16-1 and miR-15b/16-2 are sufficient to restrict the accumulation of antigen-specific T cells, Related to Figure 3.

(A,B) Frequencies of EdU^+ $CD8^+$ T cells 8 days p.i. with LCMV from single conditional knockout animals (n ≥ 6 biological replicates of 2 independent experiments, two-tailed t test). (C,D) Frequencies and absolute numbers of antigen-specific $CD8^+$ T cells 8 days p.i. with LCMV from single conditional knockout animals (n ≥ 6 biological replicates of 2 independent experiments, two-tailed t test).

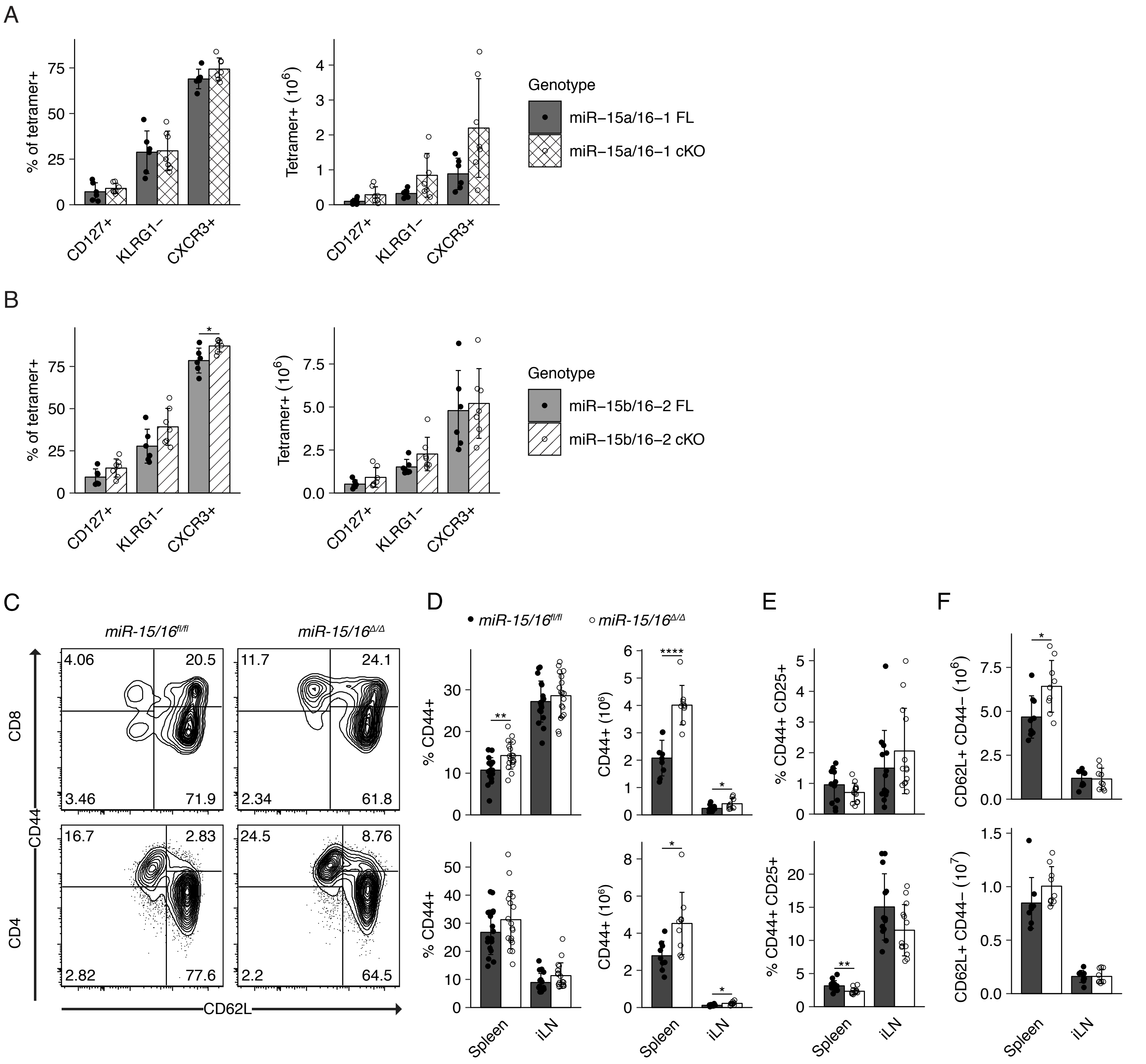

Figure S4. miR-15a/16-1 and miR-15b/16-2 are sufficient to restrict the accumulation of long-lived memory cells and miR-15/16 restrict memory cell accumulation in unchallenged animals, Related to Figure 4.

(A,B) Frequencies and absolute numbers of antigen-specific $CD8^+$ T cells 8 days p.i. with LCMV from single conditional knockout animals ($n \geq 6$ biological replicates of 2 independent experiments, two-tailed t test). (C) Flow cytometry of CD44 expression among $FOXP3^-$ $CD8^+$ (top) and $CD4^+$ (bottom) T cells within spleen and iLN collected from miR-15/16$^{fl/fl}$ and miR-15/16$^{\Delta/\Delta}$ mice. (D) Quantification of frequencies and absolute numbers of $CD44^+$ cells among $CD8^+$ (top) and $FOXP3^-CD4^+$ (bottom) T cells within spleen and iLN collected from miR-15/16$^{fl/fl}$ and miR-15/16$^{\Delta/\Delta}$ mice ($n \geq 9$ biological replicates from 3 independent experiments independent experiments, two-tailed t test). (E) Quantification of frequencies of $CD25^+$ cells among $CD44^{hi}$ $CD8^+$ (top) and $FOXP3^-CD4^+$ (bottom) T cells within spleen and iLN collected from miR-15/16$^{fl/fl}$ and miR-15/16$^{\Delta/\Delta}$ mice ($n \geq 9$ biological replicates from 3 independent experiments, two-tailed t test). (F) Quantification of absolute numbers of $CD8^+$ (top) or $FOXP3^-CD4^+$ (bottom) $CD44^-$ $CD62L^+$ T cells within miR-15/16$^{fl/fl}$ and miR-15/16$^{\Delta/\Delta}$ mice (n = 9-10 biological replicates of 2 independent experiments, two-tailed t test). *, $P < 0.05$; **, $P < 0.01$; ****, $P < 0.0001$.

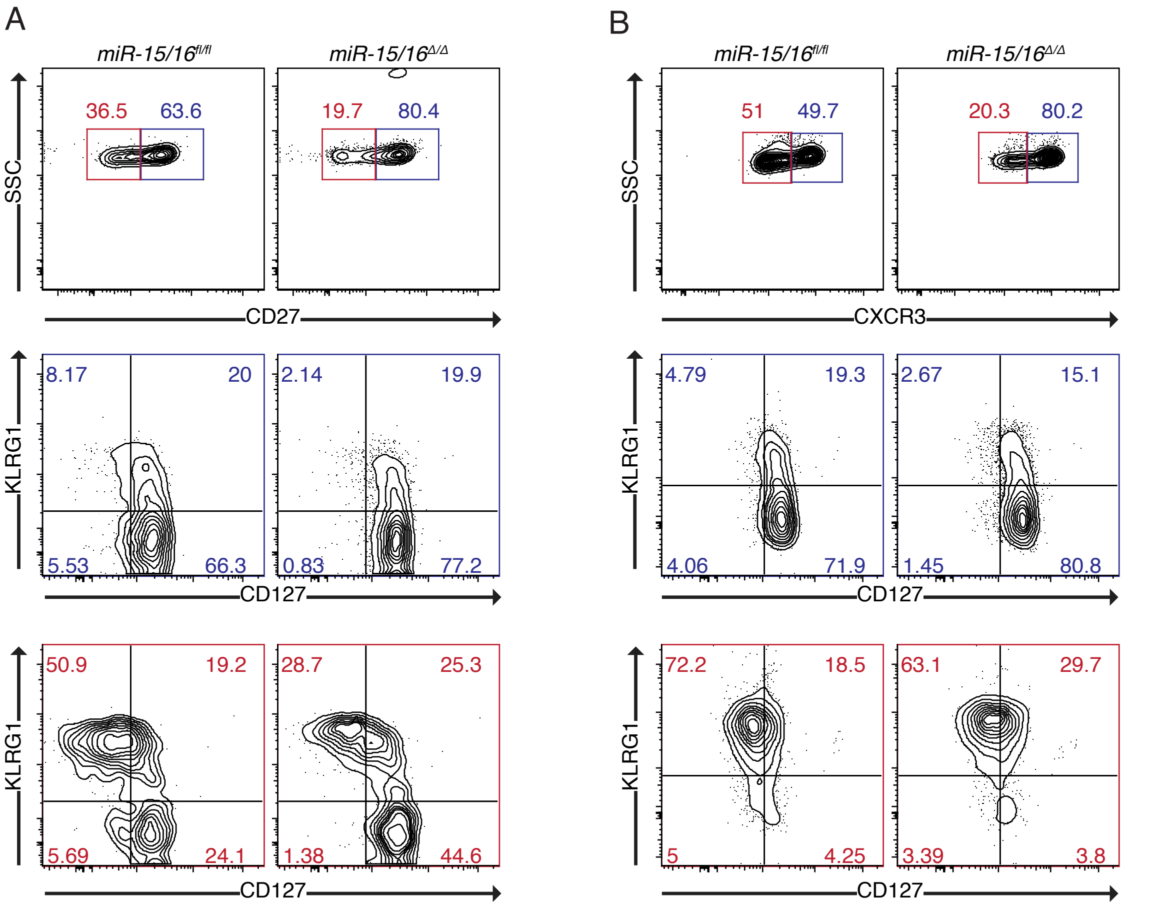

Figure S5. Integrated analysis of markers associated with memory $CD8^+$ T cells, Related to Figure 4. (A) Representative flow cytometry plots illustrating enrichment for $KLRG1^-CD127^+$ antigen-specific $CD8^+$ T cells among $CD27^+$ (B) and $CXCR3^+$.

A

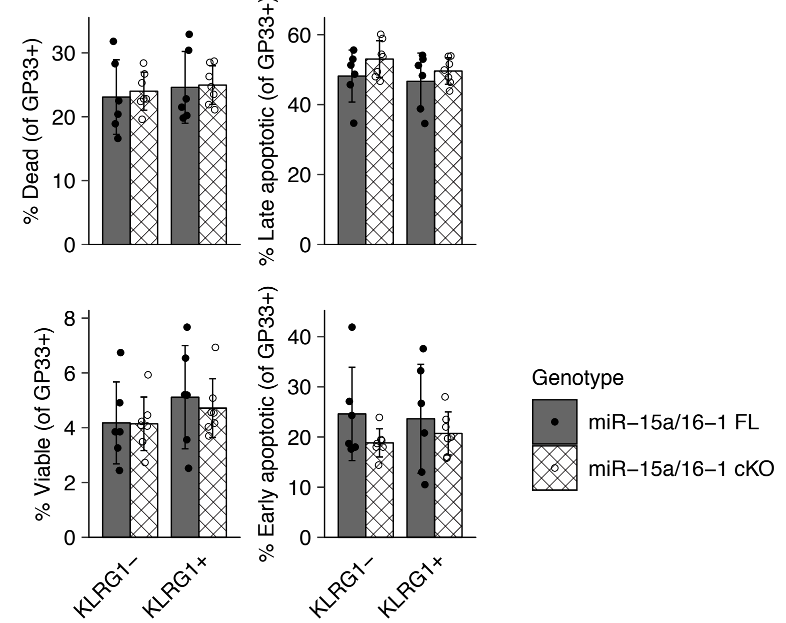

B

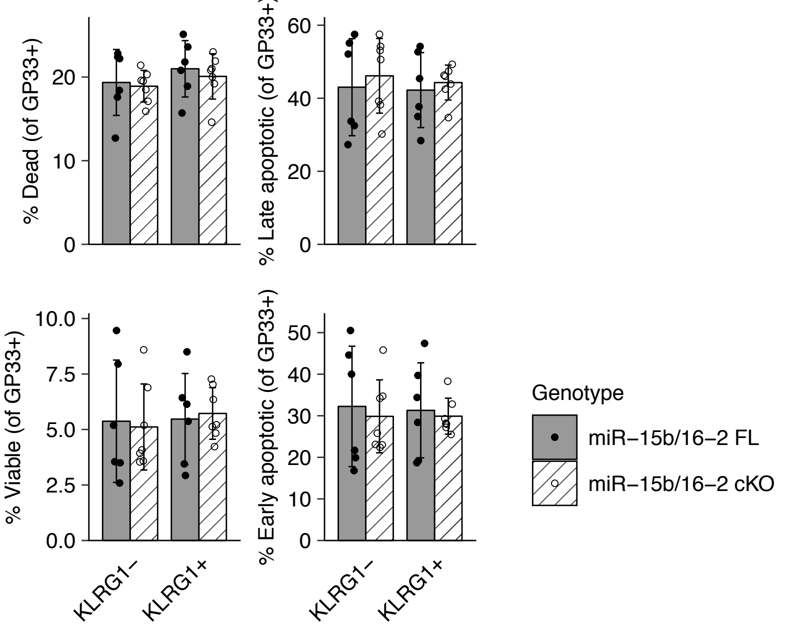

Figure S6. miR-15a/16-1 and miR-15b/16-2 are sufficient to restrict survival of antigen-specific T cells, Related to Figure 5.

(A,B) Frequencies of viable (active Caspase3$^-$ Live/Dead$^-$), early apoptotic (active Caspase3$^+$ Live/Dead$^-$), late apoptotic (active Caspase3$^+$ Live/Dead$^+$), and dead (active Caspase3$^-$ Live/Dead$^+$), antigen-specific T cells 8 days p.i. with LCMV cultured overnight in vitro from single conditional knockout animals (n ≥ 6 biological replicates of 2 independent experiments, two-tailed t test).

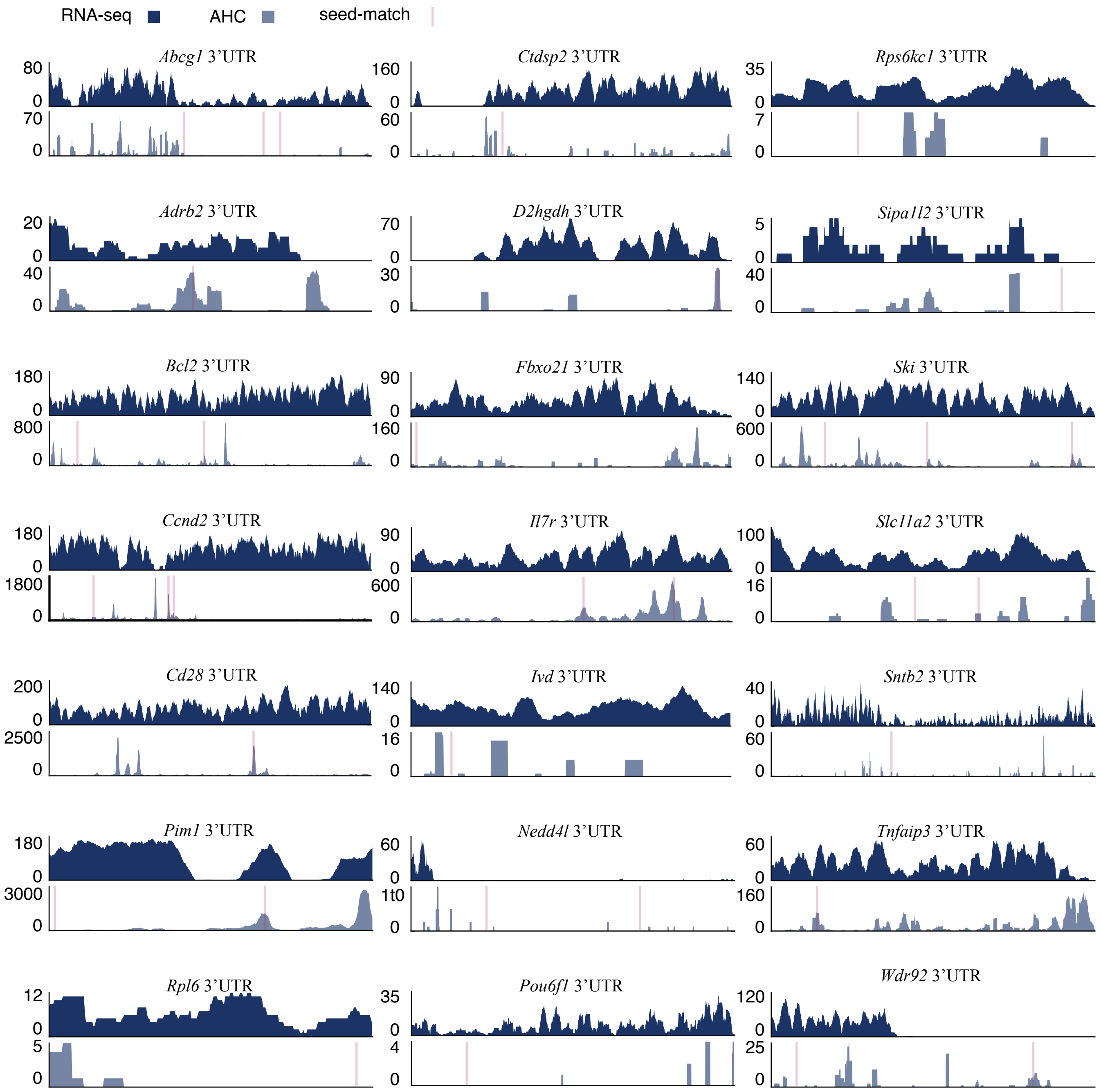

Figure S7. A network of miR-15/16 targets are up-regulated in memory cells, Related to Figure 7. RNA-seq and AHC reads aligned to the 3'UTRs of memory-associated putative targets of miR-15/16 (red shaded regions indicate locations of miR-15/16 seed-matches).
